# Field Calibration of the Optical Properties of Pedestrian Targets in Autonomous Emergency Braking Tests Using a Three-Dimensional Multi-Faceted Standard Body

**DOI:** 10.3390/s25165145

**Published:** 2025-08-19

**Authors:** Weijie Wang, Chundi Zheng, Houping Wu, Guojin Feng, Ruoduan Sun, Tao Liang, Xikuai Xie, Qiaoxiang Zhang, Yingwei He, Haiyong Gan

**Affiliations:** 1 Optics Division, National Institute of Metrology, Beijing 100029, China; wangwj@nim.ac.cn (W.W.); zhengchundi@nim.ac.cn (C.Z.); wuhp@nim.ac.cn (H.W.); fengguojin@nim.ac.cn (G.F.); sunrd@nim.ac.cn (R.S.); 19116913113@163.com (T.L.); xiexikuai@163.com (X.X.); zhangqiaoxiang@nim.ac.cn (Q.Z.); 2 College of Information Science and Technology, Beijing University of Chemical Technology, Beijing 100029, China

**Keywords:** autonomous driving, AEB target, field calibration, value transfer, BRDF

## Abstract

**Highlights:**

**What are the main findings?**

- Novel calibration apparatus: This manuscript introduces a three-dimensional multi-faceted standard body used as a value transfer medium to calibrate the optical properties (expressed as BRDF values) of pedestrian test targets. This approach is innovative because it addresses the challenges of traditional BRDF measurement methods, especially in outdoor, real-world testing scenarios.
- Integration of imaging and algorithmic methods: The proposed method combines a camera-based analytical algorithm with imaging techniques to map and transfer calibration values from a standard white plate to complex pedestrian surfaces. This integration is shown to improve the reliability of sensor calibration on non-ideal and dynamically changing targets.

**What are the implications of the main findings?**

- Enhanced calibration reliability: The development of an SI-traceable, field-applicable calibration method fills an important gap in ensuring that pedestrian target test objects used in AEB evaluations maintain consistent and reliable optical properties. This directly influences the accuracy of sensor-based safety systems in autonomous vehicles.
- Quantitative insights into target degradation: This article documents how repeated usage (i.e., crash–scatter–reassembly cycles) affects the BRDF properties of targets—showing measurable changes such as decreased uniformity and altered reflectivity. These insights have strong implications for interpreting AEB test data and for establishing maintenance or replacement schedules for test targets.

**Abstract:**

To address the growing need for field calibration of the optical properties of pedestrian targets used in autonomous emergency braking (AEB) tests, a novel three-dimensional multi-faceted standard body (TDMFSB) was developed. A camera-based analytical algorithm was proposed to evaluate the bidirectional reflectance distribution function (BRDF) characteristics of pedestrian targets. Additionally, a field calibration method applied in AEB testing scenarios (CPFAO and CPLA protocols) on one new and one aged typical pedestrian target of the same type revealed a 21% decrease in the BRDF uniformity of the aged target compared to the new one, confirming optical degradation due to repeated “crash–scatter–reassembly” cycles. The surface wear of the aged target on the side facing the vehicle produced a smoother surface, increasing its BRDF magnitude by 25% compared to the new target and making it easily detectable by the vehicle’s perception system. This led to “reverse scoring,” a safety risk in performance evaluation, necessitating timely calibration of AEB pedestrian targets to ensure reliable test results. The findings provide valuable insights into the development of regulatory techniques, evaluation standards, and technical specifications for test targets and offer a practical path toward full-life-cycle traceability and quality control.

## 1. Introduction

In recent years, the rapid increase in the number of motor vehicles has been accompanied by a steady rise in traffic accidents. According to the Global Status Report on Road Safety 2018 [1], approximately 1.35 million people worldwide lose their lives each year due to road traffic accidents. Excessive vehicle speed is one of the primary contributing factors. When a vehicle travels at speeds below 50 km/h, the risk of fatal injury to an adult pedestrian in a collision is relatively low. However, when vehicle speed exceeds 80 km/h, the fatality risk for adult pedestrians rises significantly. Research has shown that a 5% reduction in average vehicle speed can reduce traffic-related fatalities by as much as 30% [2].

The automatic emergency braking (AEB) system is an active safety feature that can autonomously apply the brakes when an imminent collision is detected, thereby reducing vehicle speed [3,4,5]. The presence of AEB systems significantly lowers the fatality rate in traffic accidents and is now considered a key indicator in evaluating the reliability and safety of advanced driver assistance systems (ADASs). It is also an important component of autonomous driving tests [6].

Between 2018 and 2021, ISO technical committees in Europe established standardized guidelines for test targets used in various traffic scenarios. These include specifications for rear-end vehicle test targets [7], pedestrian targets [8], three-dimensional vehicle targets, and cyclist targets [9]. For example, ISO 19206-2:2018 [10] stipulates that the surface reflectivity of pedestrian targets—especially the infrared reflectivity of skin-like areas—should closely match that of real human skin, typically ranging from 40% to 60%. Additionally, variations in the reflectance of clothing materials from different viewing angles should not exceed 20%.

Among all road users, pedestrians are the most vulnerable and tend to exhibit highly unpredictable movement patterns, which pose significant challenges for AEB systems in terms of detection accuracy and decision-making [11]. To date, several studies have investigated the calibration of pedestrian targets’ radar cross-section (RCS) properties, given that the RCS serves as an effective model for representing the reflectivity characteristics of target vehicles in vehicle-to-vehicle (V2V) scenarios [12]. The China Automotive Technology and Research Center Co., Ltd. (Tianjin, China) has developed radar reflection signal calibration methods for existing AEB pedestrian targets [13]. Hunan University (Changsha, China) has established standard dimensions for adult AEB test targets based on the 50th-percentile body dimensions of Chinese adults [14]. The National Intelligent Connected Vehicle Quality Infrastructure Center of China (Shenzhen, China) has implemented a calibration scheme using triangular cones for RCS data, along with signal filtering, to establish a comprehensive RCS testing protocol [15]. The University of Michigan Transportation Research Institute (UMTRI) (Ann Arbor, MI, USA) has also proposed a strategy for calibrating the RCS of AEB targets; this method effectively validates the millimeter-wave radar response characteristics of various targets, including pedestrians, cyclists, and vehicles [16].

The surface reflectance of objects significantly affects the performance of sensors in autonomous driving systems [17,18,19]. However, the reflectance of AEB test targets is influenced by factors such as the light source direction, radiation intensity, and viewing angle. These optical scattering behaviors must be characterized using bidirectional reflectance distribution function (BRDF) parameters. For pedestrian detection, most autonomous vehicles rely on technologies such as machine vision (including both visible and infrared imaging), millimeter-wave radar, multi-sensor fusion, and convolutional neural networks [20]. In this context, since image-based perception plays a critical role in identifying vulnerable road users, this study specifically focuses on analyzing the BRDF optical characteristics of pedestrian targets used in AEB systems.

Common BRDF measurement techniques and devices include a wide variety of mainstream approaches, such as the gonioreflectometer [21,22], specular BRDF acquisition systems [23], and imaging-based systems [24]. However, these systems are often limited by the shape and size of the sample object. While they allow relatively fast BRDF data collection, this often comes at the cost of reduced accuracy. Additionally, their performance is constrained by the number and angular distribution of light sources and sensors. The National Institute of Metrology, China, has developed a hemispherical BRDF calibration system, which currently serves as the national reference for BRDF calibration. For outdoor measurements, portable BRDF devices based on drone platforms have also been developed [25,26,27].

This paper proposes an in situ BRDF measurement approach for AEB test targets. The proposed method ensures the accuracy of autonomous driving test results by enabling the rapid acquisition of BRDF data from these targets. Preliminary research on imaging techniques, combined with an algorithm for bidirectional reflectance distribution function (BRDF) analysis [28], has shown the theoretical feasibility of carrying out field calibration of the optical properties of pedestrian targets. Compared to previous work [28], this study places greater emphasis on a field-viable methodology supporting experimental evaluation. Key contributions include (1) the addition of the EMD-based matching facet calculation algorithm for metrological traceability of the BRDF and (2) light source vector refinements, where instead of calculating a single combined vector, separate synthesized light vectors of the top facets are calculated for different viewing directions, making it more adaptable to field environmental constraints. These additions establish a comprehensive methodological framework for robust field assessment.

Compared to other mainstream BRDF acquisition methods, the proposed system demonstrates notable advantages in terms of cost, scalability, and accuracy.

From a cost perspective, traditional gonioreflectometers typically cost over one million CNY (Chinese yuan), while UAV-based BRDF acquisition platforms often exceed several hundred thousand CNY. In contrast, our system is designed to be cost-effective, with a total investment of about one hundred thousand CNY, making its practical deployment more viable. In terms of scalability, gonioreflectometers are mainly confined to laboratory settings and are suitable only for standardized samples. UAV-based systems offer better field adaptability but may face limitations under complex lighting or terrain conditions. The system developed in this study is inherently modular and field-oriented, offering high adaptability to a variety of testing environments.

Regarding measurement accuracy, gonioreflectometers represent the highest laboratory-grade reference standard. UAV-based platforms, while traceable to laboratory references, are often affected by environmental fluctuations such as light. Our system, however, is designed with on-site traceability in mind, incorporating light vector decomposition and determination. This allows it to maintain a balance between high-efficiency field measurements and reliable accuracy, providing a practical and quantifiable solution for BRDF calibration in real-world AEB testing scenarios.

**Figure 1:**
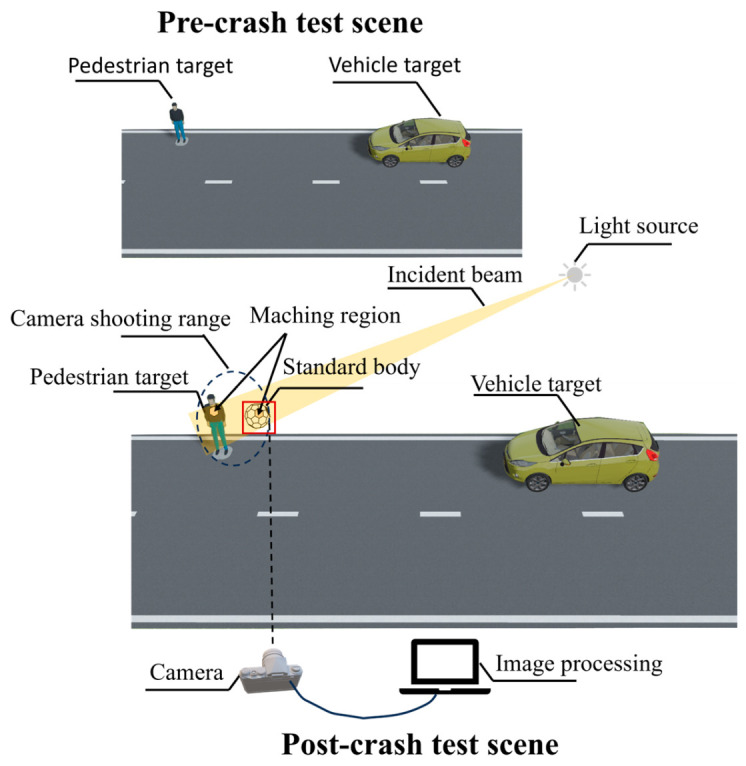
Pre- and post-crash field measurement scene.

A pre- and post-crash field measurement scene is illustrated in Figure 1. To account for the long-term use of pedestrian targets by major autonomous driving manufacturers and to ensure the representativeness and engineering comparability of the experiment, two sets of pedestrian targets were specially customized by a professional manufacturer for this study. One set consists of brand-new, unused targets serving as a reference under ideal conditions; the other set comprises targets that have been in regular use for over six months. After thorough coordination with the test site, the used targets were subjected to standard testing conditions to simulate long-term operational scenarios, allowing for evaluation of how changes in optical properties impact the performance of AEB systems.

To address the challenges in evaluating the optical properties of AEB test target surfaces and the current lack of field calibration methods, this study introduces a calibration method for the spatially distributed optical reflectance characteristics of typical humanoid targets. A field-traceable calibration apparatus was developed, which integrates a three-dimensional multi-faceted standard body with a camera. By combining imaging techniques with a BRDF analytical algorithm, the system enables rapid measurement and traceable quantification of BRDF values, including in regions that have sustained damage.

## 2. Calibration Principles and Procedures

**Figure 2:**
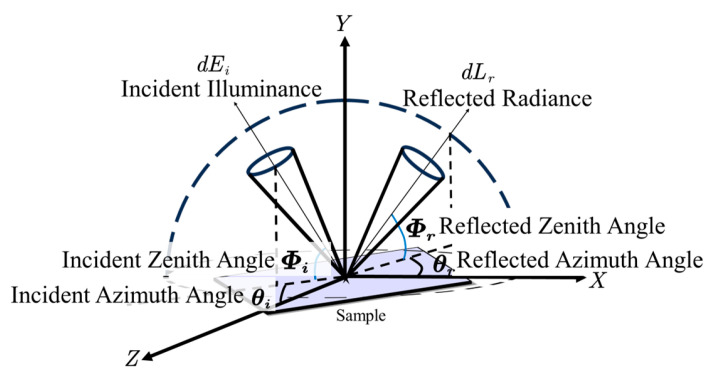
Definition of BRDF.

The bidirectional reflectance distribution function (BRDF) characterizes the scattering and radiative properties of rough surfaces. It is crucial in various applications, including target detection, tracking, recognition, feature extraction, and stealth technology [29]. As illustrated in Figure 2, the BRDF is defined as the ratio of the reflected radiance, *dL_r_*(*θ_i_,Φ_i_;θ_r_,Φ_r_*), in the reflected direction (*θ_r_,Φ_r_*) within a small solid angle to the incident illuminance, *dEi*(*θ_i_,Φ_i_*), in the incident direction (*θ_i_,Φ_i_*), also within a small solid angle. This relationship is expressed by the following equation:

(1) $$f_{r}(\theta_{i},\Phi_{i};\theta_{r},\Phi_{r})=\frac{dL_{r}(\theta_{i},\Phi_{i};\theta_{r},\Phi_{r};E_{i})}{dE_{i}(\theta_{i},\Phi_{i})}[sr^{-1}].$$

This study establishes a BRDF calibration workflow for AEB targets based on value transfer. First, the BRDF of a standard white plate (WP) is calibrated in a laboratory setting to serve as a reference. A six-axis robotic arm (Mitsubishi, Nagoya, Japan) is then employed to capture multi-angle images of both the standard white plate and a three-dimensional multi-faceted standard body. This step establishes a mapping between grayscale image values and BRDF values, thereby completing the value transfer from the standard plate to the multi-faceted body.

**Figure 3:**
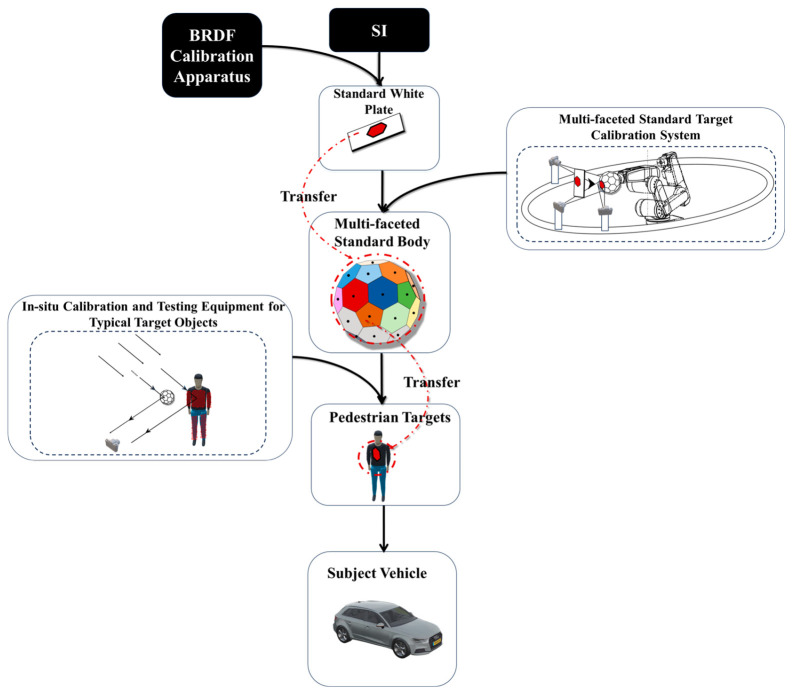
Overall calibration workflow.

Subsequently, the three-dimensional multi-faceted body is used as the transfer medium. Within a unified coordinate system, multi-angle images of the AEB target are captured. Image processing algorithms are then applied to extract vectors for the light source and viewing direction, from which BRDF values in the damaged regions of the target are derived. This enables dynamic assessment and standardized evaluation of the target’s reflective properties. The overall calibration process is illustrated in Figure 3.

### 2.1. Value Transfer Process for the Standard White Plate

The National Institute of Metrology, China, utilizes a hemispherical BRDF calibration apparatus that currently serves as the national standard for BRDF calibration. This system supports high-precision calibration of the BRDF values of standard white plates [30].

### 2.2. Value Transfer Process for the Multi-Faceted Standard Body

Once the BRDF values of the standard white plate are obtained, the multi-faceted body module undergoes BRDF calibration and value transfer in a laboratory environment. The calibration procedure is as follows.

**Figure 4:**
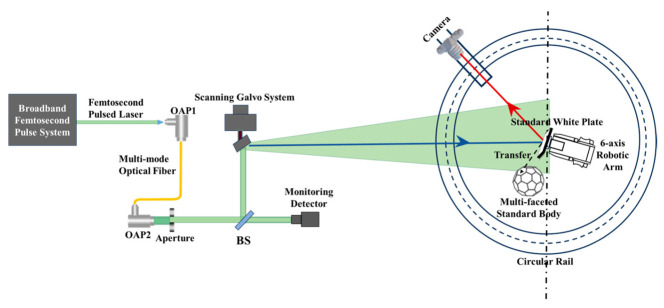
BRDF transfer process.

A specific wavelength, λ, is selected during image acquisition. The incident angle of the light source is denoted as (*θ_i_,Φ_i_*), and the camera’s viewing angle is denoted as (*θ_r_,Φ_r_*). A six-axis robotic arm is used to alternately position the standard white plate and the multi-faceted standard body for calibration. The BRDF value of the standard white plate is denoted as $f_{rWP}(\theta_{i},\Phi_{i},\theta_{r},\Phi_{r},\lambda )$. A camera is mounted on a circular rail. Using a light source with the same wavelength and maintaining the same lighting and viewing conditions as in field acquisition, images are captured from various angles. The calibration process is shown in Figure 4.

For calibration, the facet of the multi-faceted body under consideration and the corresponding facet of the standard white plate are aligned to the same position and height, thereby forming the calibration plane. The average grayscale values of the white plate and the facet to be calibrated are recorded as $L_{r\;WP}(\theta_{i},\Phi_{i},\theta_{r},\Phi_{r},\lambda )$ and $L_{r\;TDMFSB}(\theta_{i},\Phi_{i},\theta_{r},\Phi_{r},\lambda )$, respectively. The BRDF of the facet on the multi-faceted body is then calculated using the comparative method described by Equation (2):

(2) $$f_{rTDMFSB}(\theta_{i},\Phi_{i},\theta_{r},\Phi_{r},\lambda )=\frac{L_{rTDMFSB}(\theta_{i},\Phi_{i},\theta_{r},\Phi_{r},\lambda )}{L_{rWP}(\theta_{i},\Phi_{i},\theta_{r},\Phi_{r},\lambda )}\times f_{rWP}(\theta_{i},\Phi_{i},\theta_{r},\Phi_{r},\lambda ).$$

### 2.3. Transfer Process for the AEB Target

#### 2.3.1. Construction of the Measurement Scene

Field calibration must follow standardized procedures to ensure data reliability:

- Measurement Scene Selection: Priority should be given to open, unobstructed outdoor spaces. The three-dimensional multi-faceted standard body and AEB pedestrian target must be positioned away from high-reflectivity objects (e.g., metal structures or glass facades) to avoid environmental light interference. Although their spatial positions can be flexibly adjusted, an unobstructed testing field of view must be maintained to prevent occlusion in optical measurements.
- Outdoor Light Source Configuration: Natural sunlight should be used as the sole light source. Calibration should be conducted under clear weather conditions (illuminance ≥ 2000 lx), avoiding harsh midday sunlight or shadow interference.
- Indoor Light Source Configuration: A single main light source (e.g., an LED array) should be used, and all other ambient light sources must be turned off. The distance between the light source and the target must be at least five times the target length to ensure uniform illumination.
- Camera Parameter Calibration: The camera, standard body, and measurement area of the target must be at the same horizontal height. To reduce perspective distortion, the distance between the standard body and the target should be ≤*x* cm, and the distance between the camera and both the target and standard body should be ≥20*x* cm. A top-down perspective is employed during image acquisition to cover the full frontal and lateral features of the target.
- Standard Body Pre-calibration: After capturing a baseline image directly facing the camera, the camera is rotated at 90° intervals to acquire images from multiple angles. These images are used to calculate incident angles and reflected direction vectors.
- Dynamic Measurement of the Target: After fixing the incident light direction, the pedestrian target is adjusted to a predefined damage position, and images covering the full extent of the damaged region are acquired. Simultaneously, a photometer records light intensity values, sampling once per image frame.
- Dynamic Light Intensity Monitoring: For outdoor experiments, an illuminance meter is employed to monitor ambient light intensity in real time. The meter should be placed in an unobstructed area, and each image must be tagged with a corresponding light intensity timestamp for subsequent BRDF compensation calculations.

#### 2.3.2. Algorithm Processing

**Figure 5:**
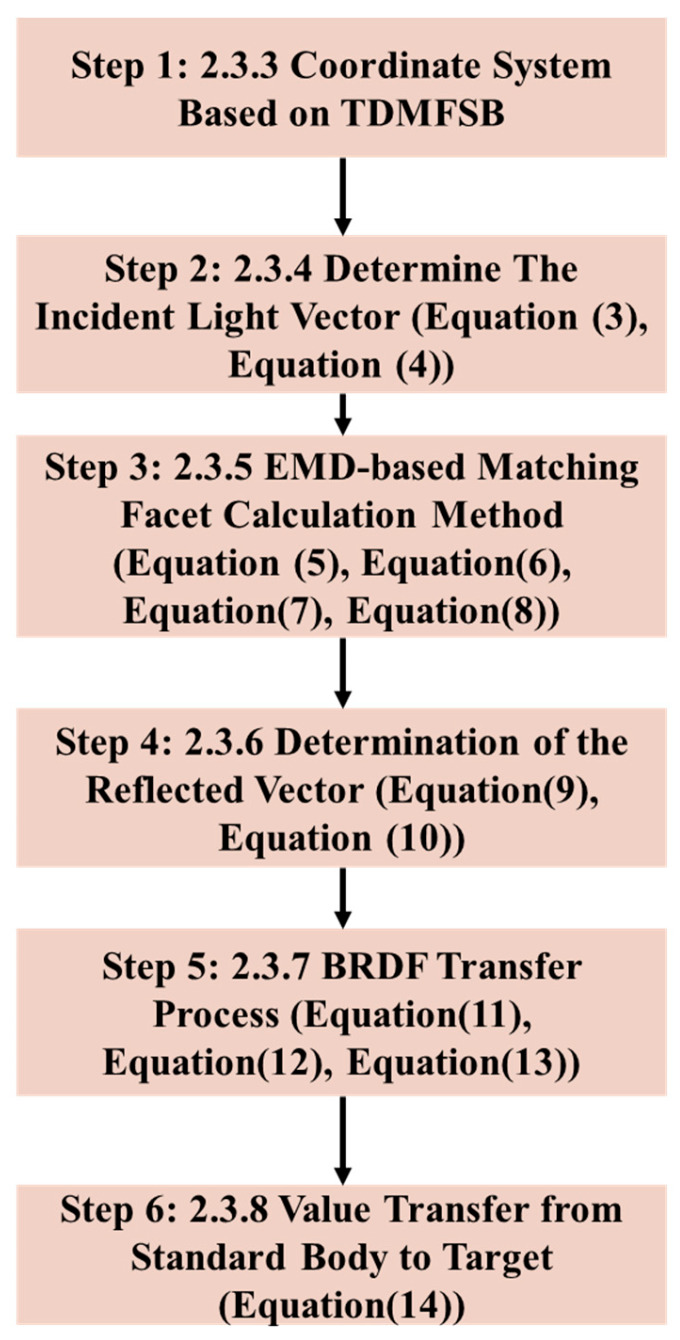
Algorithmic processing workflow.

Following image acquisition, processing proceeds through a six-step algorithmic workflow, illustrated in Figure 5.

#### 2.3.3. Coordinate System Based on TDMFSB

**Figure 6:**
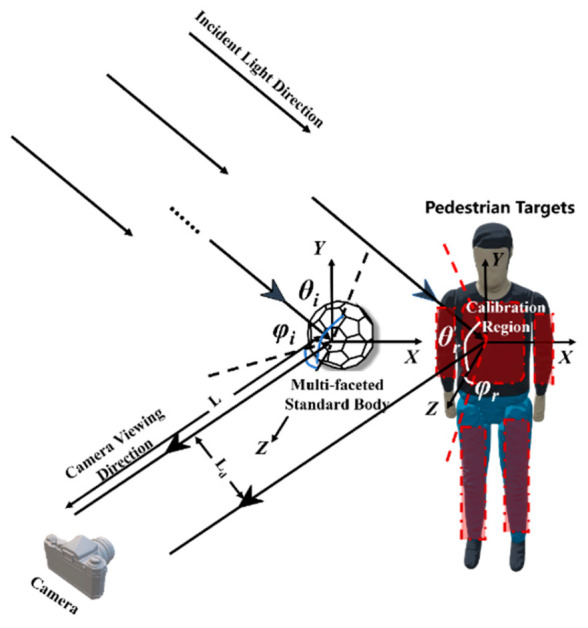
Coordinate system used. (the angular parameters *θ*_i_ and *φ*_i_ are denoted by blue lines, and the calibration region is highlighted in red).

First, the multi-faceted body module is extracted from the captured images. The outer boundary of the module is identified, and its sphere center $\left(X_{0},Y_{0},Z_{0}\right)$ is used as the origin of the coordinate system. The line connecting the sphere center and the camera center is defined as the *Z*-axis. Based on this, the coordinate system is constructed, and the radius (*R*) of the multi-faceted body module is computed. Taking the chest center of the AEB pedestrian target $\left(X_{0}^{\prime },Y_{0}^{\prime },Z_{0}^{\prime }\right)$ as the origin of the pedestrian target coordinate system, a similar coordinate system is constructed using the line connecting this point to the camera direction as the *Z*-axis. The coordinate system is illustrated in Figure 6. The distance between the AEB pedestrian target and the multi-faceted body module is denoted as *L_d_*.

#### 2.3.4. Determine the Incident Light Vector

**Figure 7:**
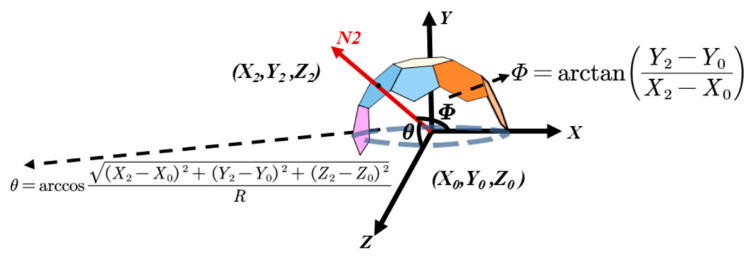
Procedure for computing polygon angles.

To determine the angular orientation of the normal vector for each polygon, four individual images of the standard body are used. For each image, the constituent polygons on the multi-faceted body module are identified, and the center coordinates of their circumscribed circles are computed. Taking the blue polygon at point (*X*_2_,*Y*_2_,*Z*_2_) in Figure 7 as an example, the center coordinate of the blue polygon (*X*_2_, *Y*_2_, *Z*_2_) represents the projection center of the polygon on the *XY* plane. Accordingly, the angle *θ* between the polygon normal and the *Z*-axis satisfies the following:

(3) $$\theta =\arccos\frac{\sqrt{(X_{2}-X_{0}{)}^{2}+(Y_{2}-Y_{0}{)}^{2}+(Z_{2}-Z_{0}{)}^{2}}}{R}.$$

The angle *Φ* between the *NZ* plane (formed by the blue polygon normal *N* and the *Z*-axis) and the *XZ* plane satisfies the following:

(4) $$\Phi =\arctan(\frac{Y_{2}-Y_{0}}{X_{2}-X_{0}}).$$

Using the same method, the angles *θ* and *Φ* can be calculated for all polygons visible in the images. Subsequently, the synthetic vector of the light source is calculated. The principle is to infer the direction from the orientation of the brightest polygons. For each polygon, the directional angles *θ* and *Φ* of its normal vector are recorded, and the average grayscale value *P_n_* in the original image is used as the magnitude of the vector within the polygon area. The resulting polar coordinate vector *N_n_* for each polygon is expressed as *N_n_* = [*θ_n_*, *Φ_n_*, *P_n_*].

From all the vectors, the one with the maximum magnitude—e.g., *N*_2_ in the figure—is identified. Then, all vectors corresponding to polygon centers with a Euclidean distance from the center (*X*_2_, *Y*_2_, *Z*_2_) of the polygon with the maximum vector *N*_2_ less than *aR* (where *R* is the radius of the multi-faceted body module) are selected—e.g., *N*_1_ through *N*_6_ in the figure. These vectors lying within the selected region are then combined to form a synthetic vector.

**Figure 8:**
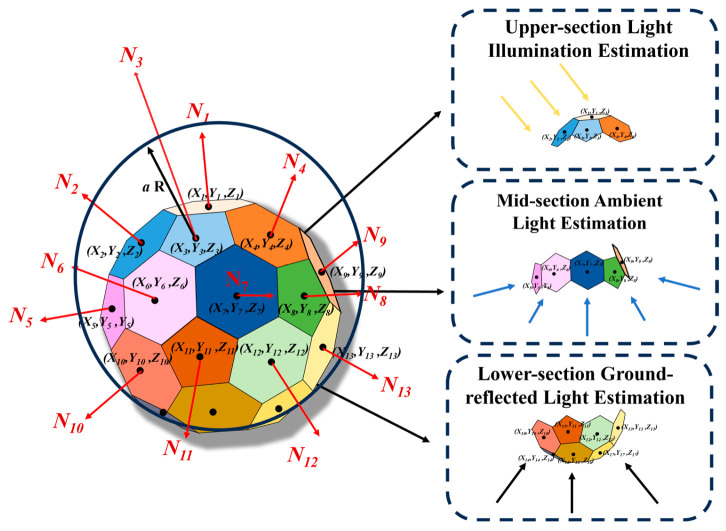
Determination of the incident vector.

The same procedure is repeated for all four images, resulting in four light source vector components. After performing coordinate transformations to unify their orientations, the four light source vectors are fused into a final synthetic incident light vector. The direction of this vector defines the incident direction of the composite light source, denoted as (*θ_i_*, *Φ_i_*). The complete procedure for determining the incident vector is shown in Figure 8.

#### 2.3.5. EMD-Based Matching Facet Calculation Method

Once the composite incident direction is determined, the next step is to identify the facet on the standard body that best matches the direction of the measured object for BRDF value transfer. The Earth Mover’s Distance (EMD) algorithm is employed for this purpose. Also known as the “bulldozer distance,” the EMD is a metric used to measure the similarity between two probability distributions [31]. The concept is analogous to reshaping terrain by moving soil—one distribution is considered a “pile of earth,” and the EMD quantifies the minimum effort required to reshape it into another. Rubner et al. [32] extended this concept to image retrieval, and it has since gained widespread academic attention:

(5) $$EMD(P,Q)=\underset{F=\{f_{ij}\}}{min}\frac{\sum_{ij}f_{ij}d_{ij}}{\sum_{ij}f_{ij}}.$$

Mathematically, the EMD measures the “transportation cost” between two point sets or distributions. For a set of size *N*, two distributions, *P* and *Q*, are defined as $S=\{(w_{j},m_{j}){\}}_{j=1}^{N}$, where *m_j_* is the *j*-th element and w_j_ is its weight. Two sets $P=\{(p_{i},u_{i}){\}}_{i=1}^{m}$ and $Q=\{(q_{j},v_{j}){\}}_{j=1}^{n}$ containing *m* and *n* elements, respectively, are given. The EMD between these sets becomes a transportation problem in which the elements of *P* act as suppliers with supplies *u_i_*, and the elements of *Q* act as consumers with demands *v_j_*. The quantities *p_i_* and *q_j_* represent supply and demand, respectively. The EMD is defined as the minimum total cost required to satisfy the supply–demand problem [33].

**Figure 9:**
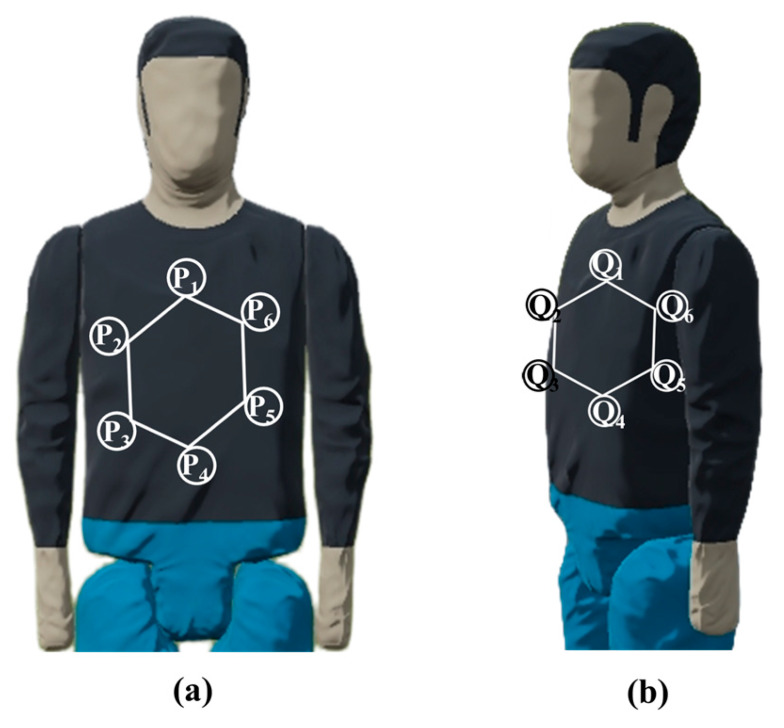
Deformation of hexagon (**a**) before and (**b**) after rotation.

To apply the EMD in the context of matching the standard body to the measured pedestrian target, a hexagon of the same size as those on the multi-faceted standard body is affixed to the test location on the target. During calibration, the target is rotated at a specific angle, which causes deformation of the hexagon, as shown in Figure 9.

**Figure 10:**
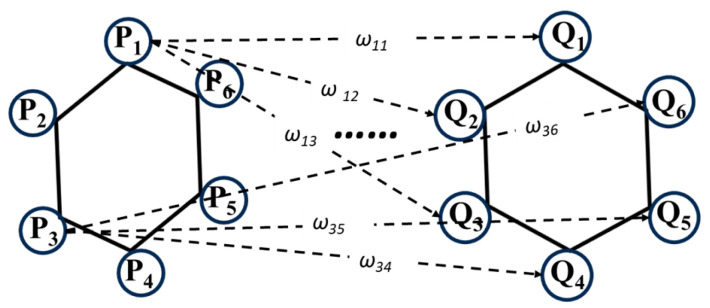
EMD computation using hexagons.

As shown in Figure 10, the pre- and post-rotation hexagons are extracted, and their vertices form two point sets, *P* and *Q*. The Euclidean distance between each vertex *P_i_* and *Q_j_* is treated as the weight *ω_ij_*.

The value *f_ij_* can be computed as follows:

(6) $$\omega_{ij}=||p_{i}-q_{j}||.$$

This yields a distance matrix *D*, where the elements *d_ij_* represent the distance between the *i*-th vertex of the base polygon *P* and the *j*-th vertex of the target polygon *Q*:

(7) $$D=|\begin{array}{cccc} \omega_{11} & \omega_{12} & \ldots & \omega_{1j}\\ \omega_{21} & \omega_{22} & \ldots & \omega_{2j}\\ \vdots & \vdots & \ddots & \vdots \\ \omega_{i1} & \omega_{i2} & \ldots & \omega_{ij} \end{array}|.$$

Assume that the computed distance matrix is

(8) $$D=[\begin{array}{ccccc} 0.5 & 1.2 & 0.9 & 1.8 & 0.7\\ 1.1 & 0.4 & 1.5 & 0.9 & 1.3\\ 0.3 & 1.0 & 0.6 & 0.8 & 1.4\\ 0.1 & 1.3 & 0.4 & 0.5 & 1.1\\ 1.0 & 0.6 & 0.8 & 0.7 & 0.2 \end{array}].$$

Using linear assignment, the optimal assignment solution yields a total cost of 0.5 + 0.4 + 0.6 + 0.5 + 0.2 = 2.2. The total mass to be transported, i.e., the number of vertices, is 5. Thus, the resulting EMD is 0.440.

In practice, a new coordinate system is established in which the hexagon on the pedestrian target and each polygon on the standard body are translated to the origin. The EMD is computed between the transformed target hexagon and every polygon on the standard body. If the number of vertices on two faces is unequal, the lower count is used as the denominator in the EMD calculation. For example, when comparing a hexagon to a pentagon, the transported mass is 5. The polygon on the standard body with the smallest EMD is selected as the matching facet (MF).

#### 2.3.6. Determination of the Reflected Vector

When capturing images directly facing the target, the line connecting the center of the sphere of the multi-faceted body module and the center of the camera is defined as the *Z*-axis. Under this condition, the viewing angle of the multi-faceted body module is (0°, 0°). Given that the distance from the camera to the target *L_r_* is significantly greater than the distance *L_d_* between the multi-faceted body module and the AEB pedestrian target, the viewing angle of the AEB pedestrian target can also be approximated as (0°, 0°).

However, when either the camera or the target is rotated by a certain angle, the camera’s angle relative to the matched facet changes and must be recalculated. If the camera is rotated by (*α*°, *β*°) after initially facing the target, the matched facet is changed to facet 5, as illustrated in Figure 6. To determine the camera’s rotation angle, the normal vector *N*_5_ of facet 5 must be rotated to align with the *Z*-axis of the coordinate system.

During this rotation, the distance between the light source and the standard body is assumed to be significantly larger than the distance from the center of the sphere to facet 5. Therefore, the light rays can be approximated as passing through both facet 5 and the sphere center simultaneously.

To construct the spatial relationship among the normal vector *N_5_* of facet 5, the light source, and the camera, three spatial distance components are introduced: *L_5_*, *L_L_*, and *L_c_*. These represent, respectively, the radius *R* of the multi-faceted body module, the distance from the light source to the module *L_L_*, and the distance from the camera to the module *L_c_*. These parameters define the spatial vectors used in the calculation.

A polar coordinate system is then established with the origin at (*X*_0_, *Y*_0_). The polar coordinates of the vectors *N_L_*, *N*_5_, and *N*_c_ are expressed as $\left(\theta_{L},\Phi_{L},L_{L}\right)$, $\left(\theta_{5},\Phi_{5},L_{5}\right)$, and (0, 0, *L_c_*), respectively. These polar coordinates are subsequently converted into Cartesian coordinates denoted as $\left(X_{L},Y_{L},Z_{L}\right)$, $\left(X_{5},Y_{5},Z_{5}\right)$, and $\left(0, 0,Z_{c}\right)$.

The rotation angles *α* and *β* can then be calculated as

(9) $$\alpha =\arctan(\frac{y_{5}}{z_{5}}),\beta =\arctan(\frac{x_{5}}{z_{5}}).$$

**Figure 11:**
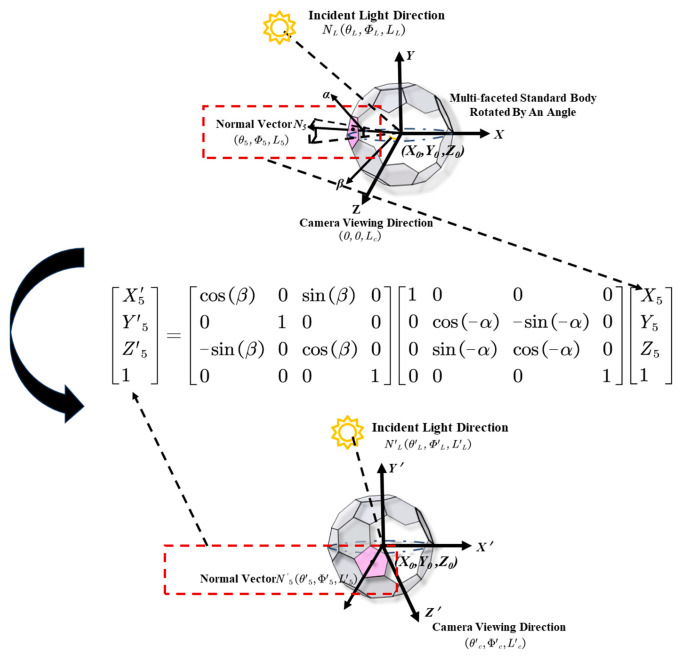
Determination of the reflected vector.

To align *N*_5_ with the *Z*-axis, it must be rotated clockwise by -*α* around the *X*-axis and clockwise by *β* around the *Y*-axis. After this transformation, facet 5 can be considered to directly face the camera, and its viewing angle is then $\left(\theta_{r},\Phi_{r}\right)$ =(α,β). This transformation demonstrates the change in the viewing angle relative to the matched facet, as shown in Figure 11.

Using coordinate transformation matrices, the rotation process can be validated. The Cartesian coordinates of the rotated normal vector *N‘_5_* can be obtained using the following equation [22]:

(10) $$[\begin{array}{c} X\prime_{5}\\ Y\prime_{5}\\ Z\prime_{5}\\ 1 \end{array}]=[\begin{array}{cccc} \cos(\beta ) & 0 & \sin(\beta ) & 0\\ 0 & 1 & 0 & 0\\ -\sin(\beta ) & 0 & \cos(\beta ) & 0\\ 0 & 0 & 0 & 1 \end{array}][\begin{array}{cccc} 1 & 0 & 0 & 0\\ 0 & \cos(-\alpha ) & -\sin(-\alpha ) & 0\\ 0 & \sin(-\alpha ) & \cos(-\alpha ) & 0\\ 0 & 0 & 0 & 1 \end{array}][\begin{array}{c} X_{5}\\ Y_{5}\\ Z_{5}\\ 1 \end{array}].$$

The accuracy of the result can be confirmed by comparing the coordinates obtained through matrix operations with those derived from algorithmic computation.

#### 2.3.7. BRDF Transfer Process

Before initiating the BRDF transfer process, it is essential to conduct a preliminary analysis of the incident lighting conditions. These conditions are influenced not only by direct solar radiation but also by scattered and reflected light emanating from atmospheric and environmental sources. Direct sunlight, as the primary light source, is characterized by strong directionality and high intensity. However, as it passes through the atmosphere, it undergoes Rayleigh and Mie scattering, resulting in diffuse illumination. This phenomenon is particularly pronounced in the short-wavelength blue region and constitutes a significant component of atmospheric scattered light [34].

Additionally, environmental surfaces, such as the ground, buildings, and vegetation, reflect sunlight and thereby act as secondary light sources. These are collectively referred to as ambient reflected light. The combined effects of direct sunlight, atmospheric scattering, ambient reflection, and cloud scattering result in complex and variable illumination conditions. Consequently, light arrives from all directions under virtually all weather conditions.

Tian et al. [35] from Peking University (Beijing, China) proposed that the BRDF (bidirectional reflectance distribution function) of vegetation canopies can be expressed as the sum of single and multiple scattering components. In practice, light often strikes a surface from various directions, and the contributions of the BRDF components vary accordingly. Therefore, the overall BRDF of a surface can be modeled as a weighted sum of the BRDF values from all incident directions.

**Figure 12:**
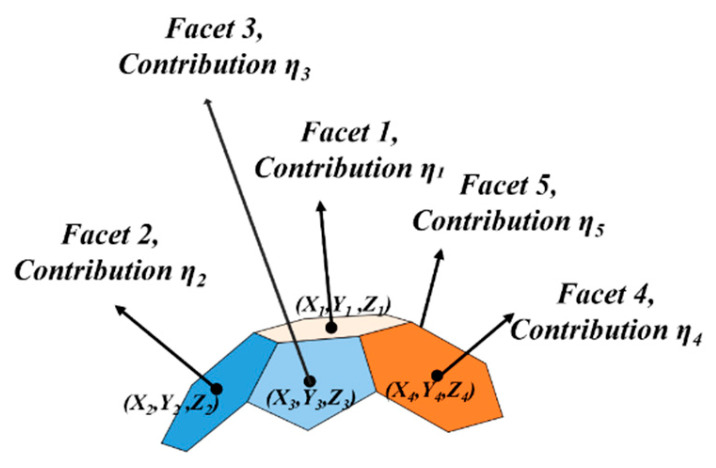
Schematic of BRDF synthesis.

Similarly, as illustrated in Figure 12, the BRDF of a matching facet on a multi-faceted standard body can be approximated as a weighted sum of the BRDFs of its individual facets. However, during field calibration, only facets 1 through 5 are considered in calculating the total incident light vector. The remaining facets are primarily illuminated by light reflected from the ground and surrounding walls. As a result, the BRDF contribution for facet 1 is mainly derived from facets 1 to 5.

The weight of each contributing facet is calculated based on the ratio of its grayscale value to the total grayscale value of all contributing facets.

Using a comparative method, the BRDF value of the calibration facet on the multi-faceted standard body is calculated as follows:

(11) $$\begin{array}{c} f_{rTDMFSB}(\theta_{i},\Phi_{i},\theta_{r},\Phi_{r},\lambda )=\\ \frac{L_{rTDMFSB}(\theta_{i},\Phi_{i},\theta_{r},\Phi_{r},\lambda )/E_{TDMFSBa}}{L_{rWPa}(\theta_{i},\Phi_{i},\theta_{r},\Phi_{r},\lambda )/E_{WPa}}\times f_{rWPa}(\theta_{i},\Phi_{i},\theta_{r},\Phi_{r},\lambda ) \end{array}.$$

where $f_{rWP}(\theta_{i},\Phi_{i},\theta_{r},\Phi_{r},\lambda )$ is the BRDF of the standard white plate under various incident light angles; $L_{r\;WP}(\theta_{i},\Phi_{i},\theta_{r},\Phi_{r},\lambda )$ is the average grayscale value of the white plate captured by the camera; $L_{rTDMFSB}(\theta_{i},\Phi_{i},\theta_{r},\Phi_{r},\lambda )$ is the average grayscale value of the multi-faceted standard body surface; and *E_TDMFSB a_* and *E_WP a_* are the ambient illuminance values during the measurements of the multi-faceted standard body and the white plate, respectively.

Compared to Equation (2), Equation (11) introduces two additional parameters, *E*_TDMFSB a_ and *E*_WP a_, which represent the ambient illuminance values measured during the imaging of the multi-faceted standard body and the white plate, respectively. In practice, these parameters reflect the stability of the light source illuminance at the calibration position, as indicated by the readings from the monitoring detector. The aim of including these two parameters is to correct for fluctuations in light source intensity that may affect the grayscale values in the process.

During image capture, the grayscale value recorded by the camera not only reflects the surface reflectance of the object but is also significantly influenced by the prevailing lighting conditions. Without compensating for illumination variation, BRDF values obtained from the same surface at different times may exhibit systematic errors.

Therefore, in Equation (11), grayscale values are normalized by their corresponding illuminance values to eliminate the influence of lighting variation, thereby enhancing the accuracy and repeatability of the BRDF calculation.

(12) $$\eta_{i}=\frac{\frac{L_{TDMFSD\;a}}{E_{TDMFSB\;a}}}{\sum_{a=1}^{5}\frac{L_{TDMFSB\;a}}{E_{TDMFSB\;a}}}.$$

The BRDF value $f_{rMF}(\theta_{i},\Phi_{i},\theta_{r},\Phi_{r},\lambda )$ of the matching facet is then given by

(13) $$f_{rMF}(\theta_{i},\Phi_{i},\theta_{r},\Phi_{r},\lambda )=\eta_{i}\times f_{rTDMFSBa}(\theta_{i},\Phi_{i},\theta_{r},\Phi_{r},\lambda ).$$

#### 2.3.8. Value Transfer from Standard Body to Target

After determining the BRDF of the matching facet on the standard body, a field image is loaded, and the grayscale values $L_{MF}$ and $L_{MeF}$ of the matching facet and the measured facet (MeF) on the pedestrian target are extracted. The BRDF of the target surface is then calculated using the following formula:

(14) $$f_{rMeF}(\theta_{i},\Phi_{i},\theta_{r},\Phi_{r},\lambda )=f_{rMF}(\theta_{i},\Phi_{i},\theta_{r},\Phi_{r},\lambda )\frac{L_{MeF}}{L_{MF}}.$$

## 3. Experimental Procedure

This section begins by presenting the experimental validation of the light vector algorithm’s effectiveness under laboratory optical darkroom conditions (Section 3.1). Subsequently, Section 3.2 and Section 3.3 detail the field implementation methodology for pedestrian target characterization based on the theoretical framework established in Section 2, including the development of a comprehensive field calibration workflow. Section 3.4 quantitatively examines how the target’s optical properties influence AEB test outcomes.

### 3.1. Indoor Light Vector Algorithm Experiments

Preliminary validation of the algorithm was conducted in a laboratory of the National Institute of Metrology, Beijing, China. The experimental setup included a three-dimensional multi-faceted standard body, a camera, and two standard white plates.

The multi-faceted standard body was fabricated via three-dimensional printing. To mitigate light leakage at the seams of the polygonal panels and enhance edge definition, black adhesive tape (Deli, Ningbo, China) was applied along the edges of each polygon.

The camera used in the experiment was a monochrome camera (Thorlabs, Newton, NJ, USA), featuring a 5-megapixel resolution and a maximum frame rate of 35 fps. The two standard white plates (NIM, Beijing, China) were made of ceramic; white plate 1 measured 10 cm × 10 cm, and white plate 2 had a diameter of 15 cm.

The verification experiment was divided into two parts. The first part aimed to validate the accuracy of the light source calculation algorithm. This experiment was conducted in a darkroom located in the underground laboratory at the Changping campus of the National Institute of Metrology, Beijing, China. The multi-faceted standard body was positioned at a height of *H* above the ground (in this experiment, the height from the ground to the chest area of the pedestrian target was 1.5 m; to ensure consistency with subsequent field experiments, the height *H* of the multi-faceted standard body above the ground was also set to 1.5 m) and at a distance of *L*_c_ from the camera (in this experiment, due to the limited size of the laboratory space, *L*_c_ = 3 m). This configuration was recorded as position 1. Subsequently, the camera was moved *L*_c_ to the left and right of the multi-faceted body, denoted as positions 2 and 3, respectively.

**Figure 13:**
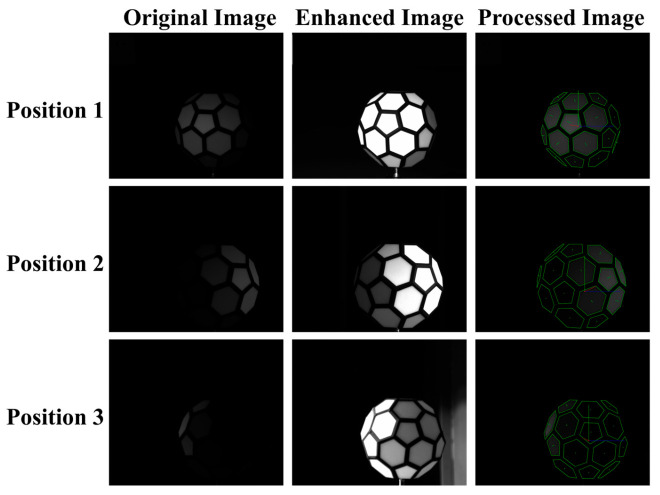
Algorithm-processed images from the verification phase.

**Figure 14:**
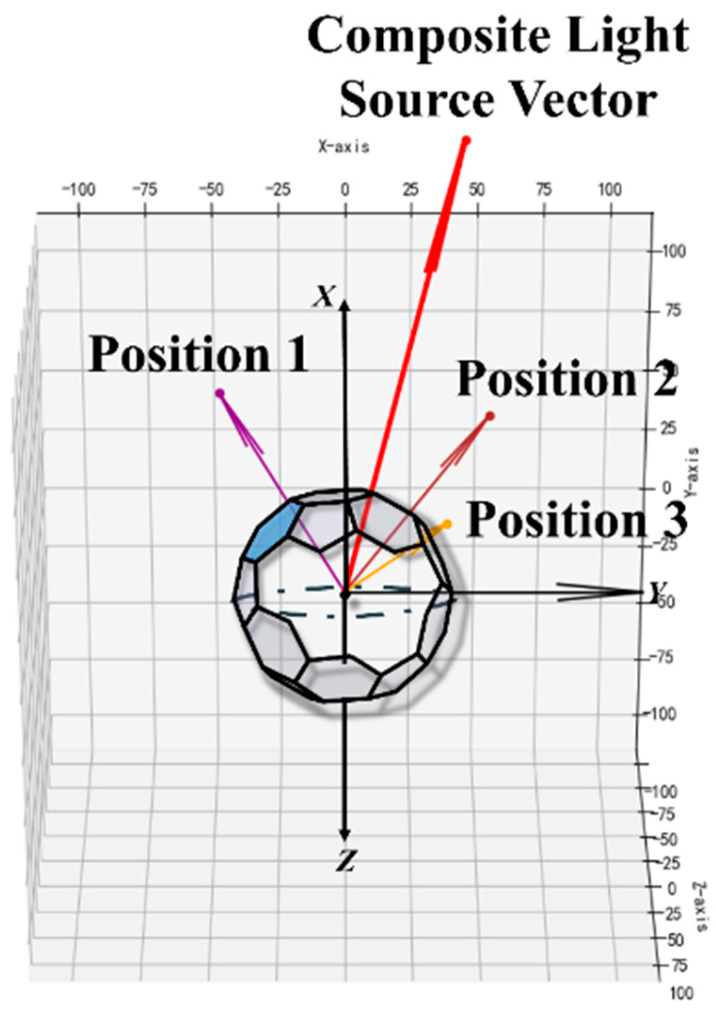
Synthetic light source vector.

The results of the algorithmic processing are shown in Figure 13 and Figure 14.

**Figure 15:**
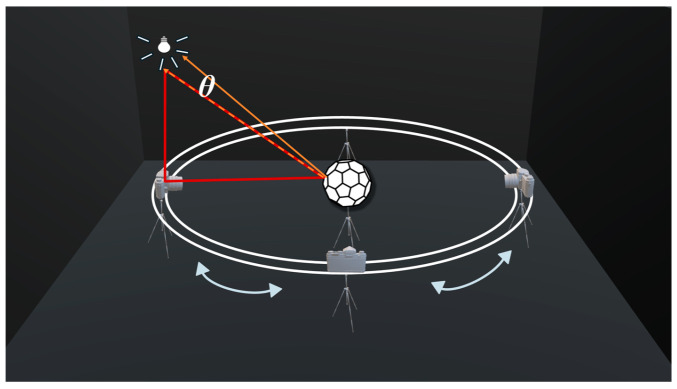
Verification of indoor light source vector.

With the reference direction established, the actual incident angle of the light source relative to the multi-faceted body was measured at (69.95°, 29.05°). The angle calculated using the synthetic light source vector was (70.76°, 30.01°). The automated detection and analysis yielded a deviation of less than *θ* (*θ* < 3°), as illustrated in Figure 15.

It should be noted that the synthesized incident direction may be influenced by refracted light from surrounding objects. As a result, there may be a certain degree of deviation between the calculated and actual incident angles.

### 3.2. Outdoor Field Testing

**Figure 16:**
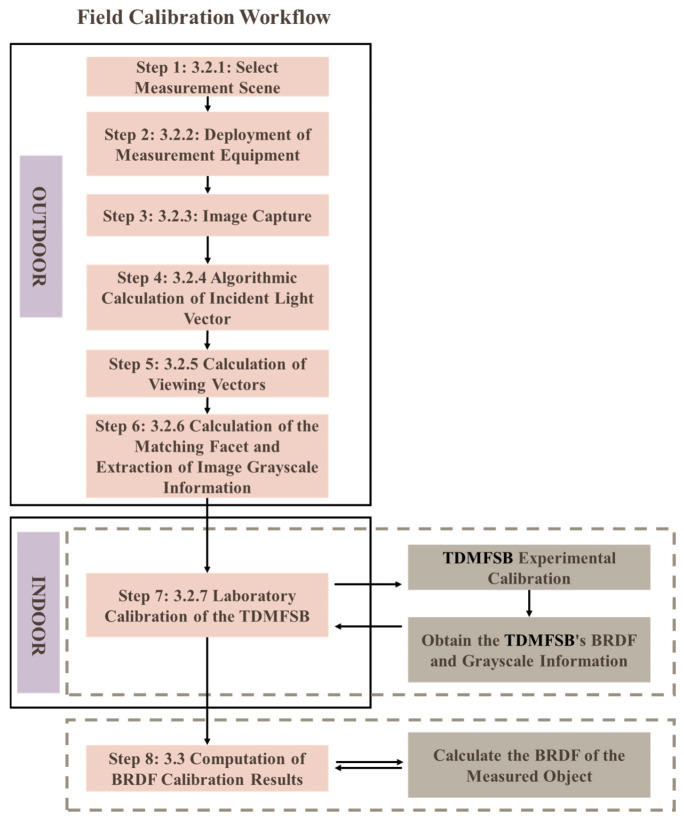
Field calibration workflow.

Based on the algorithm described in the previous section, on-site BRDF transfer for the target can be achieved. The overall field calibration workflow is illustrated in Figure 16.

Field validation experiments were conducted at the testing grounds of an institute related to intelligent connected vehicles. Two typical pedestrian targets were selected for evaluation: a new pedestrian target and an aged pedestrian target. The experimental setup included a monochrome camera equipped with a 550 nm bandpass optical filter, an Archimedean multi-faceted standard body, and the pedestrian targets. The three-dimensional multi-faceted standard body was fabricated through three-dimensional printing. The calibration process strictly followed the field calibration procedure illustrated in Figure 16.

The testing procedure consisted of the following seven steps.

#### 3.2.1. Selection of the Measurement Scene

Test scenes were selected based on the CPFAO and CPLA scenarios defined in the C-NCAP standards [36,37]:

1. A pedestrian crossing from behind an obstacle while the vehicle proceeds straight.
2. A pedestrian walking longitudinally in front of a vehicle moving straight.

These scenarios established the vehicle speed and crash points for the subsequent full-scale tests.

#### 3.2.2. Deployment of Measurement Equipment

**Figure 17:**
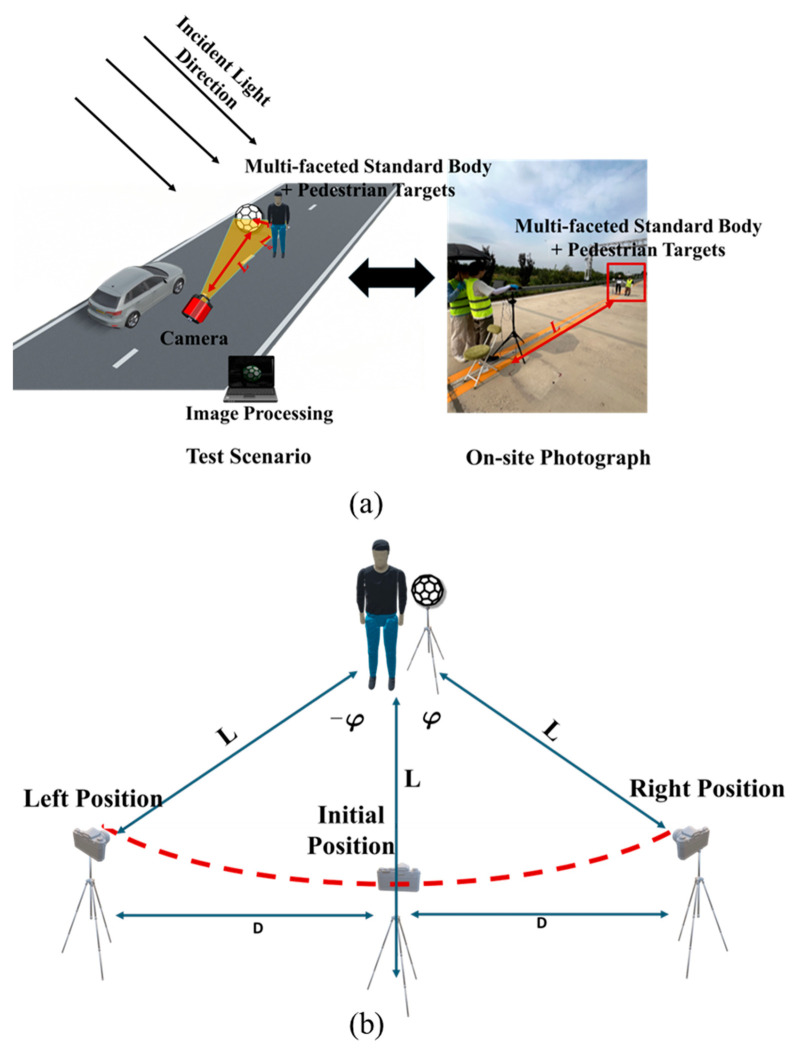
Field test scene: (**a**) layout of on-site equipment; (**b**) camera positions.

Following the layout of the measurement scene, the equipment was deployed accordingly. To capture the images required for light source calculation, the multi-faceted standard body was placed along the road centerline to avoid shadow occlusion from the surrounding background. The camera was first positioned facing direction 1 at a distance of *L*_c_ from the standard body (to ensure consistency with the calibration procedure conducted in the laboratory, the distance was also set to *L*_c_ = 3 m), and this location was marked as position 1. Subsequently, the camera was placed at positions 2 and 3, facing directions 2 and 3, respectively, each at the same distance from the standard body, and images were taken at each position. The field experiment scene is illustrated in Figure 17.

**Figure 18:**
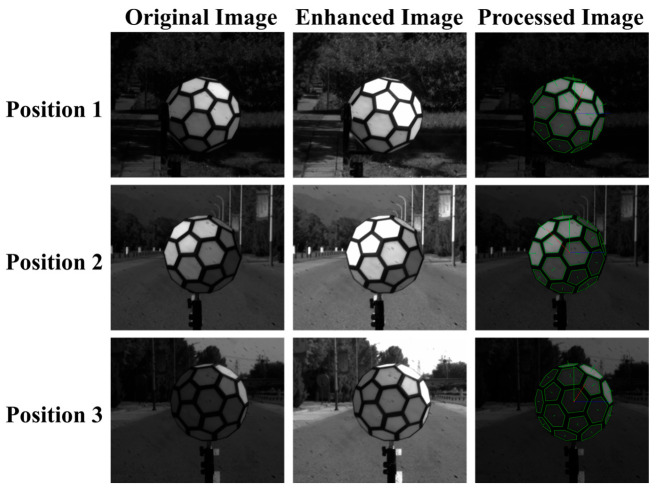
Images captured during the light source vector calculation phase.

For BRDF value transfer imaging, the pedestrian target was positioned *L*_d_ cm to the right of the standard body (in this experiment, *L*_d_ = 10 cm). Three camera positions were established: position 1 was located *L* meters directly perpendicular to the line connecting the target and the standard body (in this experiment, due to the relevant provisions in ISO-19206, *L* = 10 m); positions 2 and 3 were placed 3 m to the left and right of position 1, respectively. These positions provided a viewing angle range of −16.7° to 16.7°. Positions 2 and 3 were slightly adjusted forward to maintain a constant 10 m distance from the target. Images captured during the light source vector calculation phase are illustrated in Figure 18.

#### 3.2.3. Image Capture

After setting up the equipment, image acquisition commenced. The pedestrian target was first oriented facing the camera. Three images were taken at position 1, and the illuminance readings at the time of imaging were recorded. The target was then rotated to face sideways and backwards, and images were captured for each orientation in the same manner. After completing the sequence at position 1, the camera was moved to positions 2 and 3, and the process was repeated. The images were collected during the entire calibration process.

#### 3.2.4. Algorithmic Calculation of Incident Light Vector

Following image capture, the images from the light source vector calculation phase were processed. Since the upper portion of the standard body receives the strongest illumination and the least environmental reflection, only the upper incident vector was computed. Using the algorithm, facet-normal vectors were derived, and the corresponding polygons were filtered within a circular region with a Euclidean distance from the center of the polygon with the maximum vector of less than *aR* (with *a* set to 0.8 in this experiment), establishing the coordinate system. Within this system, the zenith and azimuth angles were defined as (*θ*_1_, *φ*_1_) = (−10.1°, 79.9°). The remaining facets were processed in a similar manner.

#### 3.2.5. Calculation of Viewing Vectors

The camera was placed at three different positions. Within the defined coordinate system, the viewing angle was expressed in terms of *θ*_r_ and *φ*_r_. Because the camera moved only within the *XZ* plane, *φ_r_* remained at 0°, while *θ*_r_ took on three values:

- *θ_r_*_1_ = −16.7° (left view);
- *θ_r_*_2_ = 0° (frontal view);
- *θ_r_*_3_ = 16.7° (right view).

Following the above steps, the incident and viewing angles for facets 1 through 5 were determined. The angular data are summarized in Table 1.

#### 3.2.6. Calculation of the Matching Facet and Extraction of Image Grayscale Information

**Figure 19:**
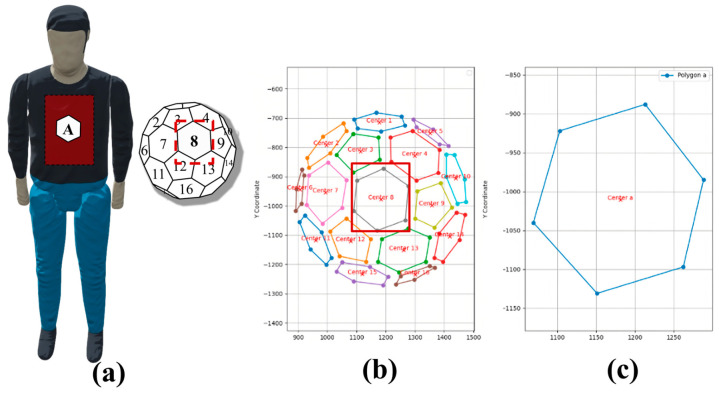
The EMD matching process: (**a**) partial matching regions between the target and the standard body; (**b**) all facets of the standard body involved in EMD computation; (**c**) the computed matching facet.

The matching facet between the multi-faceted standard body and the pedestrian target was determined using a sample scene, in which the camera, placed at position 1, captured a frontal image of the pedestrian target. In Figure 19, the red region on the pedestrian target was approximated as a plane. Hexagonal patches were pre-applied to the target, and each patch was compared with every facet of the standard body using the Earth Mover’s Distance (EMD) algorithm to identify the best match.

For the scene with the camera positioned in front of the pedestrian target, the EMD values between surface A on the pedestrian target and all facets of the standard body were calculated using Equation (5). These results are presented in Table 2. Comparison of the values revealed that facet 8 of the standard body had the smallest EMD value (22.91) relative to surface A. Therefore, facet 8 was identified as the matching facet for the approximate frontal region of the pedestrian target.

Subsequently, the average grayscale values *L_MF_* and *L_MeF_* of the approximate region on the pedestrian target and its corresponding matching facet on the standard body were extracted and recorded.

#### 3.2.7. Laboratory Calibration of the TDMFSB

After obtaining the incident light vectors for facets 1 to 5, the standard body was transported to the laboratory for calibration, following the procedure described in Section 2. The laboratory setup included a six-degree-of-freedom robotic arm and a monochrome camera. Both the standard white plate and the standard body undergoing calibration were positioned 2.3 m from the camera. To match the 550 nm bandpass filter mounted on the camera lens, a broadband tunable laser radiation field source with a wavelength of 550 nm was selected to perform illumination.

In the field experiment, the incident angle of the light source was (*θ_i_*, *Φ_i_*), and the angle between the laser and the camera was denoted by *θ*. During laboratory calibration, the camera was placed in three orientations relative to the standard body: directly in front, 16.7° to the left, and 16.7° to the right. The robotic arm’s zenith angle was adjusted to simulate these camera viewing angles, as defined by *φ_r_* in Table 1.

**Figure 20:**
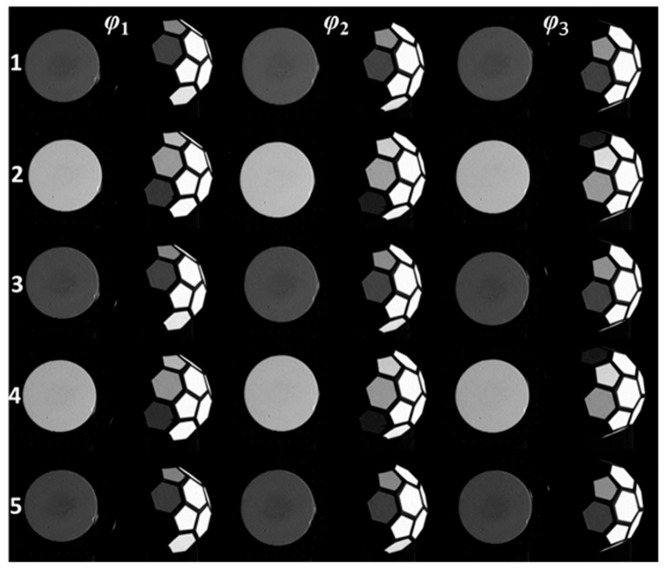
Images captured during calibration of the standard white plate and standard body.

The standard white plate and the standard body were alternately mounted on the robotic arm. At each angular position listed in Table 1, images were captured in accordance with the method described in Section 2. Illuminance readings were recorded for each image. Representative images obtained during this process are shown in Figure 20. Labels 1–5 correspond to the sources of incident light vectors; *φ*_1_, *φ*_2_, and *φ*_3_ represent the angular positions listed in Table 1, simulating the left view (arm raised), frontal view (arm level), and right view (arm lowered), respectively—corresponding to field calibration perspectives.

**Figure 21:**
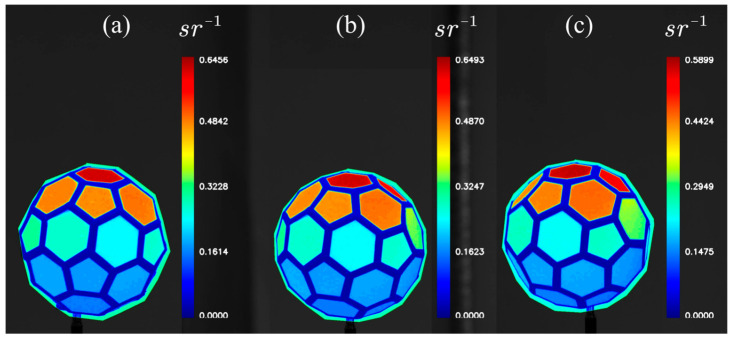
Pseudo-color visualization of BRDF results for the standard body: (**a**) BRDF visualization when the target is captured from *θ*_r1_ = −16.7°; (**b**) BRDF visualization when the target is captured from *θ*_r2_ = 0°; (**c**) BRDF visualization when the target is captured from *θ*_r3_ = 16.7°.

After image acquisition, each image set was processed to extract the grayscale values $L_{rWP}(\theta_{i},\Phi_{i},\theta_{r},\Phi_{r},\lambda )$ and $L_{rTDMFSB}(\theta_{i},\Phi_{i},\theta_{r},\Phi_{r},\lambda )$ from both the standard white plate and the corresponding facet of the standard body. Using Equation (12), the synthetic bidirectional reflectance distribution function (BRDF) value for the matching facet of the standard body was calculated. The resulting BRDF values were visualized using pseudo-color processing applied to monochrome images of the standard body. The results are displayed in Figure 21.

### 3.3. Computation of BRDF Calibration Results

After obtaining the BRDF values for each facet of the standard body through laboratory calibration, the final BRDF values of the pedestrian target were computed. This was achieved by combining the calibrated BRDF values with the average grayscale values of the matching and measured facets, using the equations previously described. Both regional averaging and pixel-wise computation approaches were employed.

**Figure 22:**
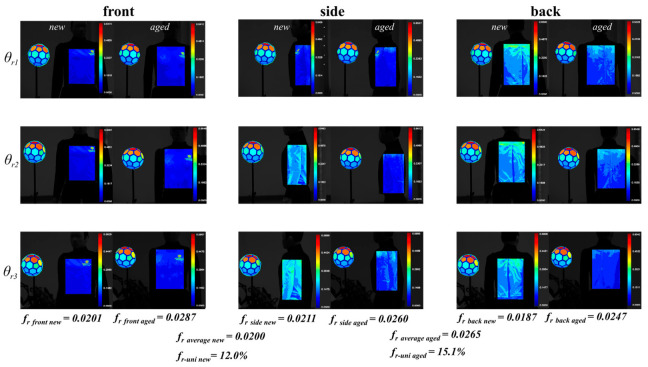
BRDF calibration and pseudo-color visualization results for new and aged pedestrian targets (front, side, back views).

Calibration was conducted from three camera positions—front, side, and back—for each orientation of the pedestrian target, resulting in nine sets of data. The computed BRDF results were then visualized using pseudo-color rendering. The visualization outcomes are presented in Figure 22. The calculation process for the quantities labeled in the figure refers to Section 3.4.3.

### 3.4. Correlative Analysis of Pedestrian Target Characteristics and AEB Performance in Autonomous Driving

Following the BRDF calibration of the pedestrian targets, a series of full-scale vehicle tests were performed to evaluate AEB (autonomous emergency braking) performance. These tests, which assessed targets with differing characteristics, were conducted in accordance with the CPFAO and CPLA test scenarios outlined in the C-NCAP protocol. CPFAO (car-to-pedestrian farside adult with obstruction) refers to a scenario in which the vehicle collides with a farside adult pedestrian target under an obstruction condition. In this scenario, the sensors of the vehicle under test primarily detect the front and side information of the pedestrian target. CPLA (car-to-pedestrian longitudinal adult) refers to a pedestrian target walking longitudinally in the vehicle’s path. In this scenario, the sensors of the vehicle under test (VUT) primarily detect the rear-side information of the pedestrian target.

**Figure 23:**
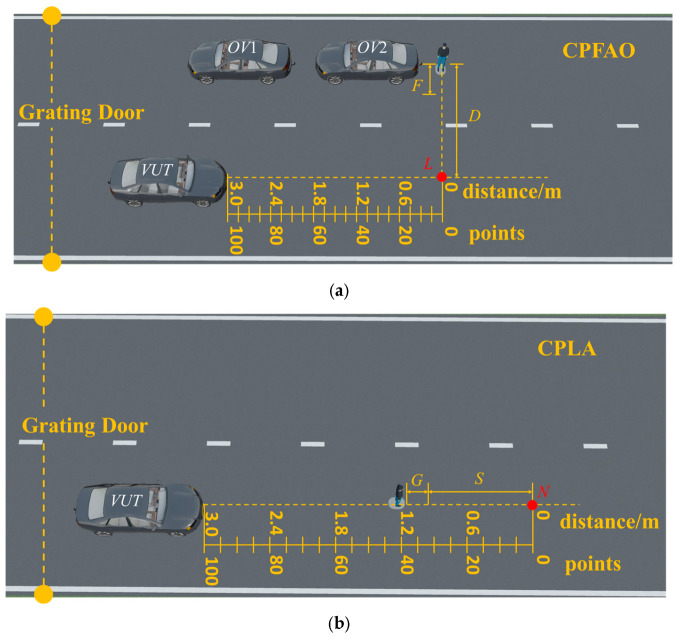
Test scenes: (**a**) CPFAO and (**b**) CPLA. *F*: the acceleration distance of the target; *D*: the distance from the target’s starting point to the theoretical crash point; point *L*: the 25% crash point on the front structure of the test vehicle; *G*: the acceleration distance of the pedestrian target; *S*: the constant-speed walking distance of the pedestrian target; point *N*: the 50% crash point on the front structure of the test vehicle; *VUT*: vehicle under test; *OV*: obstacle vehicle.

In Figure 23a, F stands for the acceleration distance of the target; D stands for the distance from the target’s starting point to the theoretical crash point; L stands for the 25% crash point on the front structure of the test vehicle. In Figure 23b, G stands for the acceleration distance of the pedestrian target; S stands for the constant-speed walking distance of the pedestrian target; and N stands for the 50% crash point on the front structure of the test vehicle. In addition, a grating door is the trigger device for the pedestrian target’s traction system, designed to synchronize the timing between the vehicle under test (VUT) and the pedestrian target. The position of the grating door varies with the vehicle speed to ensure that when the VUT passes through the grating door, the traction system begins to move the pedestrian target. Without any braking intervention by the VUT, this setup enables a collision to occur between the vehicle and the pedestrian target at the anticipated crash point.

Scoring criteria were established to assess the performance of intelligent vehicles, providing a standardized framework for evaluating their perception capabilities. As shown in Figure 23, the CPFAO and CPLA scoring systems are based on the distance between the vehicle’s stop point and the theoretical crash point. A score of 0 was assigned if the stop point was within 0–15 cm of the crash point. The score increased by 5 points for every additional 15 cm: a score of 5 for 15–30 cm, a score of 10 for 30–45 cm, and so on. A maximum score of 100 was awarded when the stop point exceeded 3 m from the theoretical crash point. The scoring system is summarized in Table 3.

Collision avoidance tests were conducted using a typical pedestrian target, considered a vulnerable road user (VRU), in both the CPFAO and CPLA scenarios as defined by C-NCAP, using a range of vehicle speeds.

In the CPFAO scenario, the test vehicle accelerated from a standstill to a constant speed of 10 km/h, 20 km/h, or 30 km/h. The pedestrian target crossed the street perpendicularly at a constant speed of 6.5 km/h, emerging from behind an obstacle on the far side of the road (opposite the driver’s side).

In the CPLA scenario, the test vehicle maintained a constant speed of 10 km/h, 15 km/h, or 20 km/h, while the pedestrian target moved longitudinally at 6.5 km/h. The target began 25 m ahead of the vehicle and moved in the same direction.

In both scenarios, once the vehicle’s sensors detected the pedestrian target, the AEB system was activated. The system first issued a warning and then automatically initiated emergency braking in an effort to avoid a collision. During each test, the response time following AEB activation and the final stop position of the vehicle were precisely recorded. A theoretical crash point—defined as the collision location assuming no evasive action by the pedestrian and no braking by the vehicle—was established. The distance between the actual stop point and the theoretical crash point was then measured.

**Figure A1:**
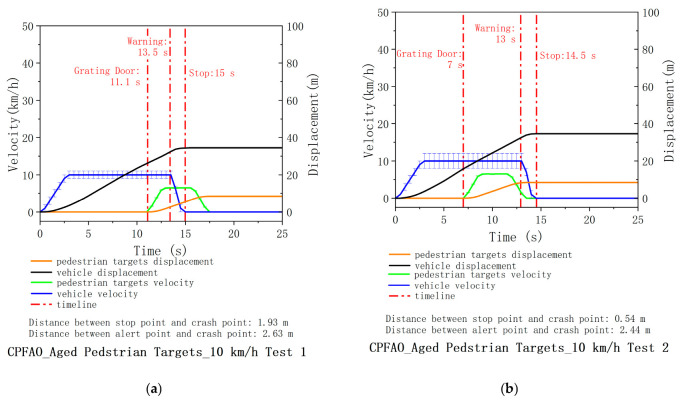

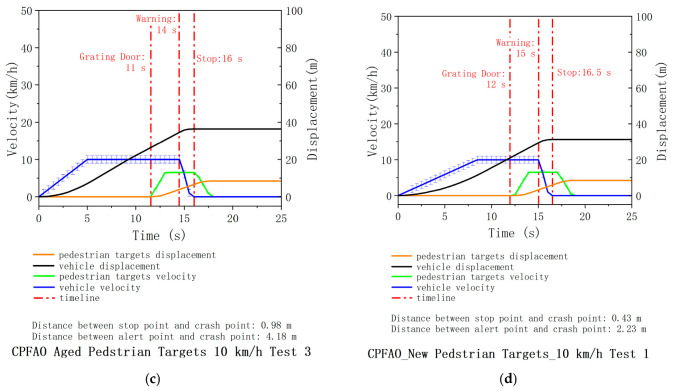
CPFAO—10 km/h test results: (**a**) CPFAO aged pedestrian target 10 km/h test 1; (**b**) CPFAO aged pedestrian target 10 km/h test 2; (**c**) CPFAO aged pedestrian target 10 km/h test 3; (**d**) CPFAO new pedestrian target 10 km/h test 1.

**Figure A2:**
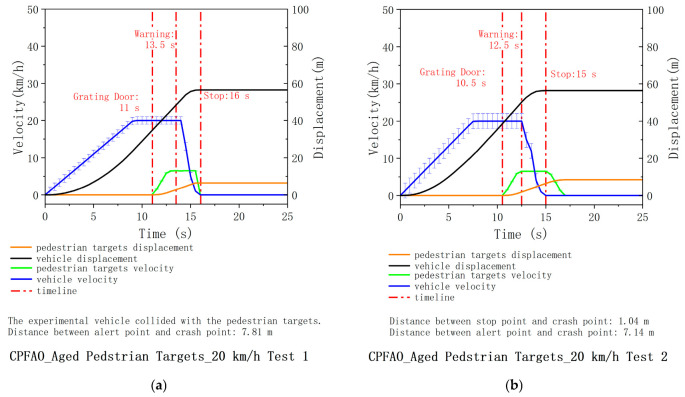

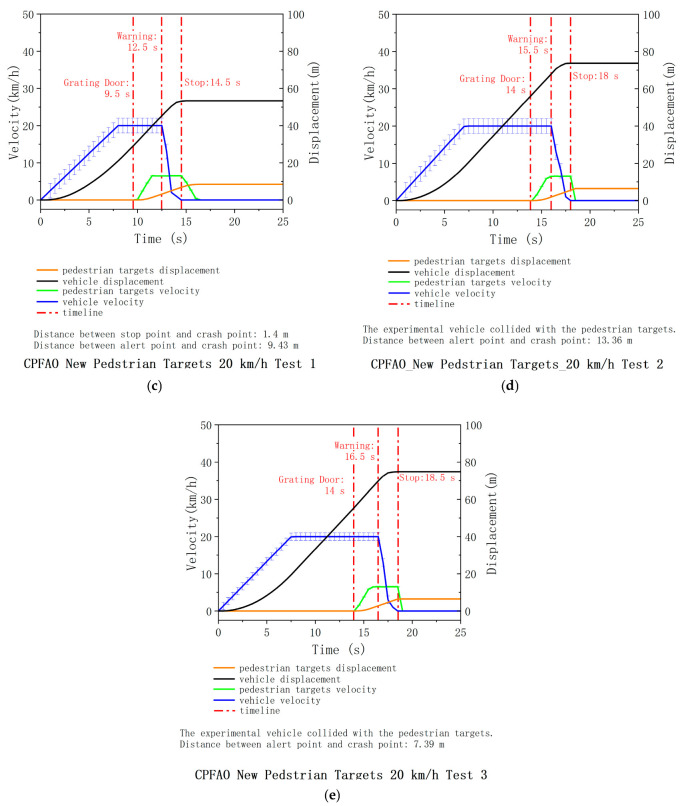
CPFAO—20 km/h test results: (**a**) CPFAO aged pedestrian target 20 km/h test 1; (**b**) CPFAO aged pedestrian target 20 km/h test 2; (**c**) CPFAO new pedestrian target 20 km/h test 1; (**d**) CPFAO new pedestrian target 20 km/h test 2; (**e**) CPFAO new pedestrian target 20 km/h test 3.

**Figure A3:**
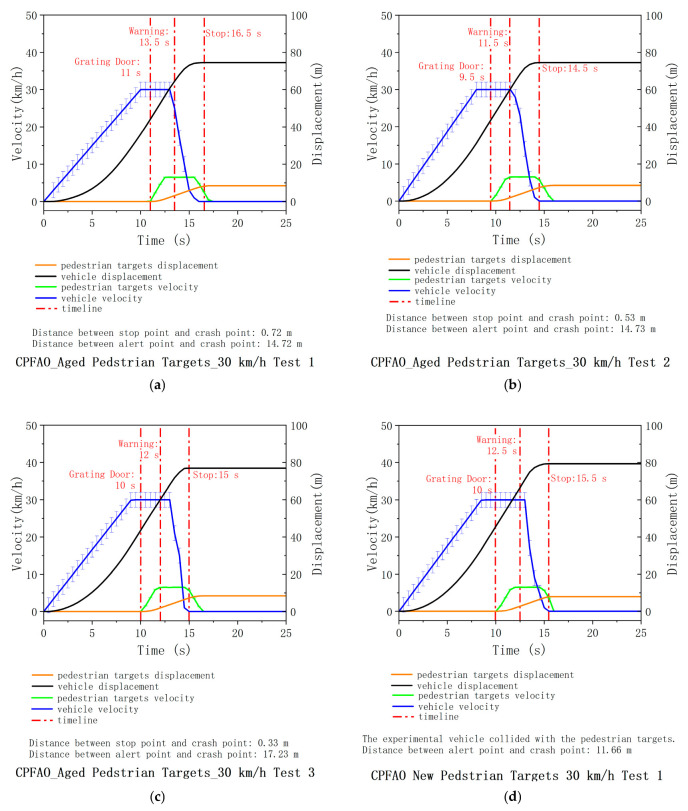

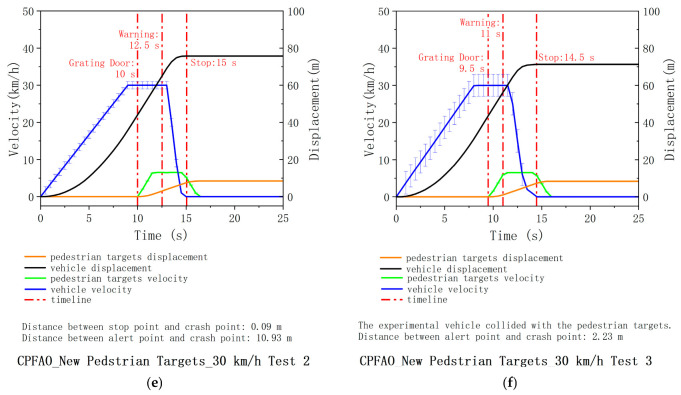
CPFAO—30 km/h test results: (**a**) CPFAO aged pedestrian target 30 km/h test 1; (**b**) CPFAO aged pedestrian target 30 km/h test 2; (**c**) CPFAO aged pedestrian target 30 km/h test 3; (**d**) CPFAO new pedestrian target 30 km/h test 1; (**e**) CPFAO new pedestrian target 30 km/h test 2; (**f**) CPFAO new pedestrian target 30 km/h test 3.

**Figure A4:**
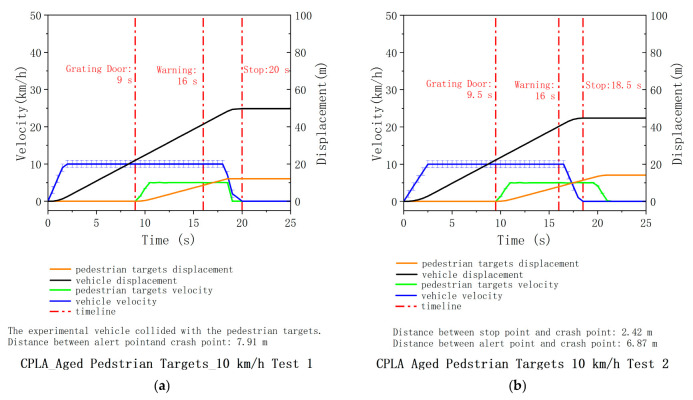

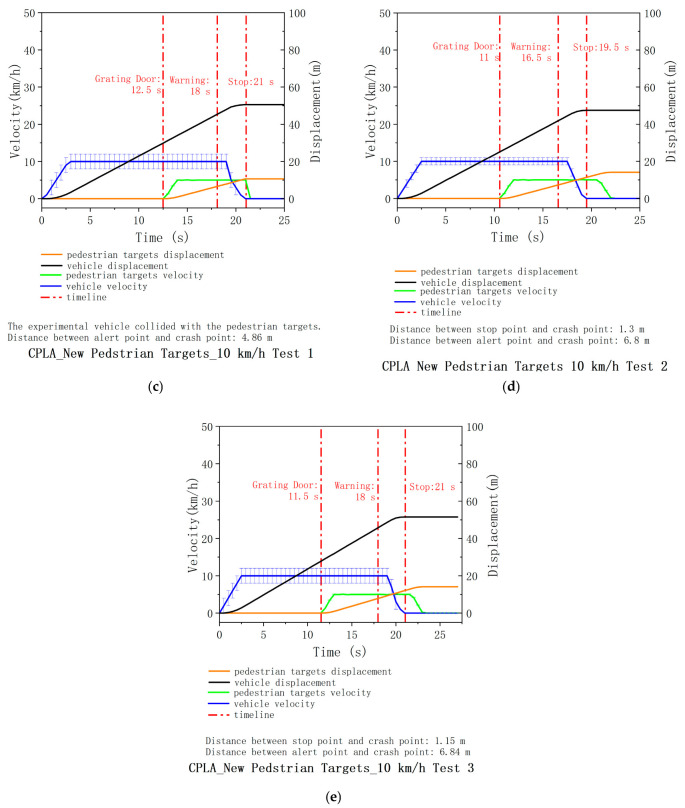
CPLA—10 km/h test results: (**a**) CPLA aged pedestrian target 10 km/h test 1; (**b**) CPLA aged pedestrian target 10 km/h test 2; (**c**) CPLA new pedestrian target 10 km/h test 1; (**d**) CPLA new pedestrian target 10 km/h test 2; (**e**) CPLA new pedestrian target 10 km/h test 3.

**Figure A5:**
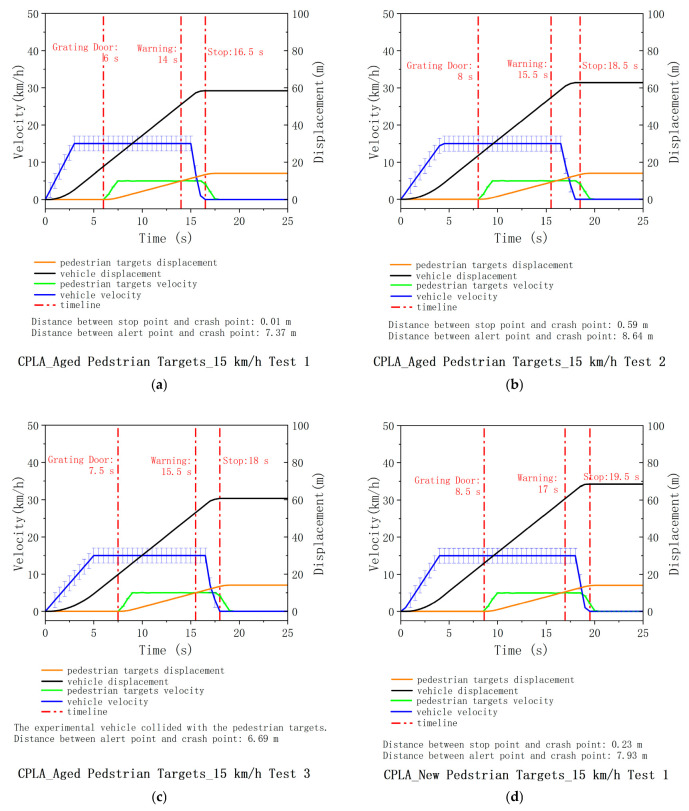

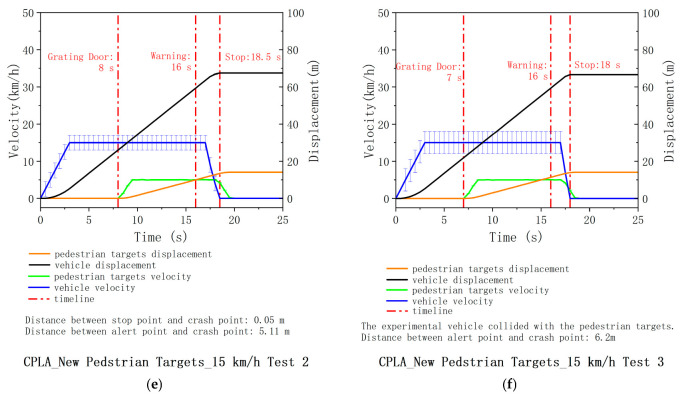
CPLA—15 km/h test results: (**a**) CPLA aged pedestrian target 15 km/h test 1; (**b**) CPLA aged pedestrian target 15 km/h test 2; (**c**) CPLA aged pedestrian target 15 km/h test 3; (**d**) CPLA new pedestrian target 15 km/h test 1; (**e**) CPLfA new pedestrian target 15 km/h test 2; (**f**) CPLA new pedestrian target 15 km/h test 3.

**Figure A6:**
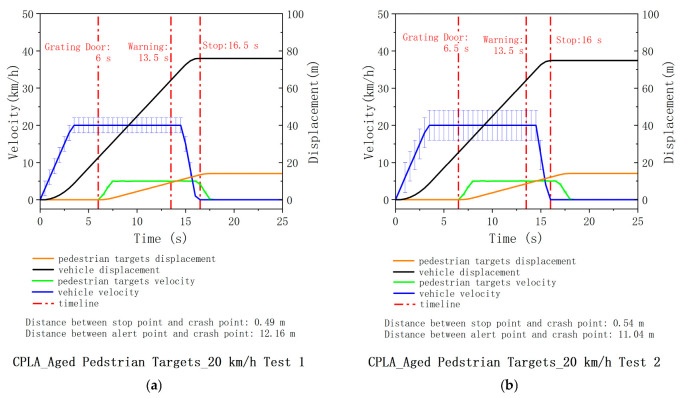

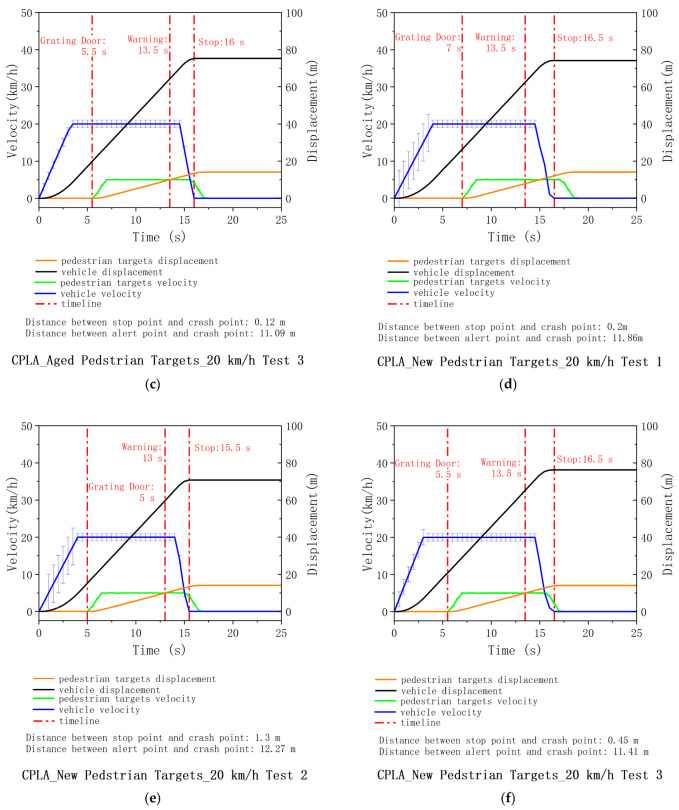
CPLA—20 km/h test results: (**a**) CPLA aged pedestrian target 20 km/h test 1; (**b**) CPLA aged pedestrian target 20 km/h test 2; (**c**) CPLA aged pedestrian target 20 km/h test 3; (**d**) CPLA new pedestrian target 20 km/h test 1; (**e**) CPLA new pedestrian target 20 km/h test 2; (**f**) CPLA new pedestrian target 20 km/h test 3.

For each vehicle speed, three collision test trials were conducted. The displacement and velocity profiles of various objects were derived from the experiments and are presented below. Figure A1, Figure A2, Figure A3, Figure A4, Figure A5 and Figure A6 of Appendix A display the experimental data obtained at different vehicle speeds in the CPFAO and CPLA test scenarios.

#### 3.4.1. CPFAO Test Results

Tests were conducted in the CPFAO scenario at vehicle speeds of 10 km/h, 20 km/h, and 30 km/h, with the following data collected.

(1) CPFAO—10 km/h

In the CPFAO scenario, real vehicle tests were conducted at a speed of 10 km/h using the two types of pedestrian targets. The experimental results are shown in Figure A1. The average collision distance for the aged pedestrian target was 1.15 m, while that for the new pedestrian target was 0.43 m.

(2) CPFAO—20 km/h

In the CPFAO scenario, real vehicle tests were conducted at a speed of 20 km/h using the two types of pedestrian targets. The experimental results are shown in Figure A2. The average collision distance for the aged pedestrian target was 1.04 m, while that for the new pedestrian target was 1.4 m.

(3) CPFAO—30 km/h

In the CPFAO scenario, real vehicle tests were conducted at a speed of 30 km/h using the two types of pedestrian targets. The experimental results are shown in Figure A3. The average collision distance for the aged pedestrian target was 0.53 m, while that for the new pedestrian target was 0.09 m.

The scoring results for the CPFAO test scenario are presented in Table 4.

#### 3.4.2. CPLA Test Results

Tests were conducted in the CPLA scenario at vehicle speeds of 10 km/h, 15 km/h, and 20 km/h, with the following data collected.

(1) CPLA—10 km/h

In the CPLA scenario, real vehicle tests were conducted at a speed of 10 km/h using the two types of pedestrian targets. The experimental results are shown in Figure A4. The average collision distance for the aged pedestrian target was 2.42 m, while that for the new pedestrian target was 0.85 m.

(2) CPLA—15 km/h

In the CPLA scenario, real vehicle tests were conducted at a speed of 15 km/h using the two types of pedestrian targets. The experimental results are shown in Figure A5. The average collision distance for the aged pedestrian target was 0.30 m, while that for the new pedestrian target was 0.14 m.

(3) CPLA—20 km/h

In the CPLA scenario, real vehicle tests were conducted at a speed of 10 km/h using the two types of pedestrian targets. The experimental results are shown in Figure A6. The average collision distance for the aged pedestrian target was 0.38 m, while that for the new pedestrian target was 0.65 m.

The scoring results for the CPLA test scenario are presented in Table 5.

#### 3.4.3. BRDF vs. Score Data Analysis

The results above were summarized based on the vehicle scores in both test scenarios. First, the BRDF calibration values of the pedestrian targets were evaluated. For each target, the average BRDF values for the front, side, and back are denoted as *f*_rfront_*, f*_rside_*,* and *f*_rback_, respectively.

For the CPFAO scenario, in which the pedestrian crosses the street, the combined average of the front and side BRDF values was used as the representative calibration value, denoted as *f*_r(CPFAO)_. In the CPLA scenario, where the vehicle follows the pedestrian, the back BRDF value was used as the representative value, denoted as *f*_r(CPLA)_. The overall average BRDF value across all facets of a pedestrian target is denoted as *f*_ravg_, and the relative standard deviation of this average is represented as *f*_r-uni_.

The computational formula is given by

(15) $$f_{r-uni}=\frac{f_{rmax}-f_{rmin}}{f_{ravg}}.$$

Based on the calculation using Equation (15), the BRDF uniformity values *f*_r-uni(aged)_ and *f*_r-uni(new)_ for the aged and new pedestrian targets are 15.1% and 12.0%, respectively, indicating a 21% relative difference in uniformity between the two targets. In the CPFAO scenario, the approaching vehicle primarily observes the front and side surfaces of the pedestrian target. The average BRDF values of the front and side surfaces, denoted as *f*_r(CPFAO)aged_ and *f*_r(CPFAO)new_, are 0.0274 and 0.0206, respectively. This indicates that the aged target has a 25% higher average BRDF than the new target in these directions. Similarly, in the CPLA scenario, where the vehicle approaches from behind, the observed surface is the back of the pedestrian target. The average BRDF values of the back surfaces, denoted as *f*_r(CPLA)aged_ and *f*_r(CPLA)new_, are 0.0247 and 0.0187, respectively. Again, the aged target exhibits a 25% higher average BRDF than the new target.

**Figure 24:**
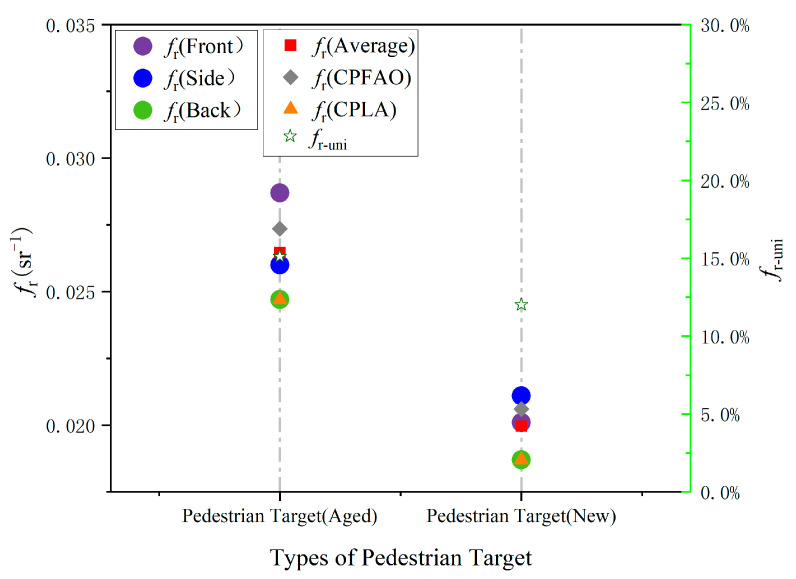
BRDF calibration results and variation ranges of pedestrian targets.

Based on the above calculation rules, the BRDF calibration results and variation ranges for the pedestrian targets are illustrated in Figure 24.

**Figure 25:**
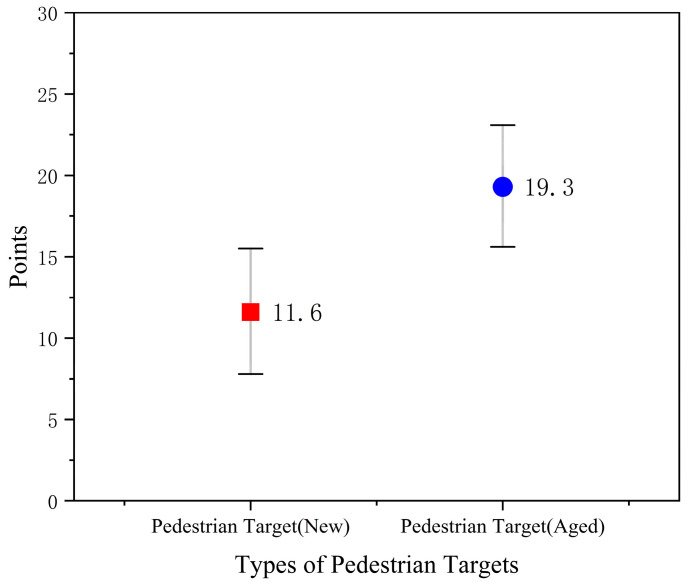
Summary of experimental scores for the two targets.

The final experimental scores for the two pedestrian targets are summarized in Figure 25. Let the test score of pedestrian target *i* (where *i* = new, aged) in test scenario *s* (where *s* = CPFAO, CPLA) during the *j*-th trial be denoted as *S*_i,s,j_. The final average score ${\overset{―}{S}}_{i}$ of each type of pedestrian target is calculated as follows:

(16) $${\overset{―}{S}}_{i}=\frac{1}{N}\sum_{s}\sum_{j=1}^{n_{s}}S_{\;i,s,j}.$$

Here, *N* represents the total number of tests conducted for each target across both scenarios, and *n_s_* denotes the number of test repetitions under scenario *s* (in this study, *n*_CPFAO_ = 9 and *n*_CPLA_ = 9; thus, *N* = 18). *S_i,s,j_* is the score of the *j*-th test. The average scores ${\overset{―}{S}}_{new}$, ${\overset{―}{S}}_{aged}$ are calculated separately for the new and aged pedestrian targets, and the results are summarized in Figure 25.

#### 3.4.4. Conclusions from Experimental Data

Based on the experimental data described above, the final conclusions are as follows:

1. In both the CPFAO and CPLA test scenarios, as vehicle speed increased, the corresponding test scores decreased. This trend indicates that overall safety performance declines with rising vehicle velocity.
2. Although the new and aged pedestrian targets belong to the same pedestrian target type, the BRDF value of the aged target in the direction of vehicle approach was approximately 25% higher than that of the new target in both test scenarios. Consequently, the aged target was more easily detected by the vehicle under the same environmental conditions, allowing the vehicle to initiate braking more effectively. This surface change increases BRDF values and enhances the target’s detectability, potentially leading to artificially improved safety performance scores during AEB testing.
3. After approximately six months of repeated use, the conformity of the aged target with ISO requirements declined by around 21% compared to that of the new target. Repeated impacts and surface abrasion significantly degraded the diffuse reflection properties of the target’s surface. The front-facing surface of the aged target became smoother, resulting in stronger reflectivity and improved detectability. This led to the observation that tests using the aged target yielded higher scores than those using the new one. This introduces a safety paradox, where degraded physical targets produce misleadingly favorable results, potentially introducing safety risks with continued usage.

## 4. Summary and Discussion

This study presents a comprehensive account of the development and application of perception testing modules and field calibration techniques for typical target objects in intelligent connected vehicles. As the technology behind intelligent connected vehicles continues to rapidly evolve, the need for reliable and robust testing methodologies has become increasingly urgent.

Among the critical safety functions of advanced driver assistance systems (ADASs), automatic emergency braking (AEB) is particularly vital. However, inconsistencies in the quality and optical reflectance characteristics of test targets have compromised the accuracy and reliability of current AEB evaluations. These limitations significantly affect the safety performance of autonomous vehicles.

To address this issue, this study introduces a field calibration method based on bidirectional reflectance distribution function (BRDF) imaging. A novel three-dimensional multi-faceted standard body was employed as a value transfer medium to enable accurate on-site calibration of the optical reflectance characteristics of typical pedestrian targets. The proposed method comprehensively evaluates target reflectance by constructing a three-dimensional coordinate system, capturing multiple images, calculating light source and viewing vectors, and deriving BRDF values.

Experimental results confirmed that the proposed method satisfies the optical reflectance requirements specified in standards such as ISO 19206. This confirmation provides strong assurance of the reliability and consistency of AEB testing.

In practical application, this study demonstrates the effectiveness of the autonomous driving perception testing module and the field calibration techniques through full-scale vehicle tests. By creating modular test scenarios that replicate a range of real-world road and traffic conditions, the study enabled a thorough evaluation of perception, decision-making, and execution capabilities in autonomous vehicles. The test results show that the developed testing module and calibration technology significantly enhance both the efficiency and accuracy of autonomous vehicle testing. This lays a strong foundation for the broader commercialization of intelligent connected vehicles.

Importantly, this study also highlights a potential safety concern in AEB testing: when the BRDF uniformity of a pedestrian target undergoes a significant negative deviation—such as increasing to 15% or 25%—it may lead to a counterintuitive phenomenon where aged targets receive higher scores than new targets, a situation referred to as “score inversion.” This outcome compromises the fairness and reliability of safety performance assessments. Therefore, it is suggested that AEB test developers and relevant stakeholders pay close attention to BRDF uniformity during testing.

To enhance the study’s practical value, a three-tiered compensation framework can be proposed: (1) short-term correction factors derived from real-time BRDF monitoring to neutralize uniformity bias; (2) medium-term replacement cycles calibrated to target degradation rates; and (3) long-term standardization of a threshold (e.g., ≤15%) of BRDF uniformity. This would help prevent misleading results and support the establishment of more robust and fair evaluation standards.

In conclusion, the development of an autonomous driving perception testing module and field calibration techniques for typical targets has effectively improved the testing accuracy and efficiency of intelligent connected vehicles. These contributions offer essential support for the commercialization of autonomous driving technologies and address a critical gap in the reliable evaluation of the optical properties of test targets. Looking ahead, as technology continues to advance and applications expand, these techniques are expected to play an increasingly pivotal role in accelerating the development of the intelligent connected vehicle industry.

## Figures and Tables

**Table 1:** Angular computation results for facets 1–5 during field and laboratory calibration stages.

Source of Light Vector	Light Vector Zenith Angle *φ*_i_	Viewing Vector Zenith Angle *φ*_r_	Viewing Vector Azimuth Angle *θ*_r1_ (Left View)	Viewing Vector Azimuth Angle *θ*_r2_ (Frontal View)	Viewing Vector Azimuth Angle *θ*_r3_ (Right View)	Angle Between Light and Camera Viewing Direction *θ*_i1_ (Frontal View)	Angle Between Light and Camera Viewing Direction *θ*_i2_ (Left or Right View)	Robotic Arm Zenith Angle *φ*_1_ (Left View)	Robotic Arm Zenith Angle *φ*_2_ (Frontal View)	Robotic Arm Zenith Angle *φ*_3_ (Right View)
Facet 1	79.9°	0°	−16.7°	0°	16.7°	79.88°	80.83°	−16.7°	0°	16.7°
Facet 2	69.1°	0°	−16.7°	0°	16.7°	69.73°	66.27°	−16.7°	0°	16.7°
Facet 3	80°	0°	−16.7°	0°	16.7°	80.06°	81.92°	−16.7°	0°	16.7°
Facet 4	69.5°	0°	−16.7°	0°	16.7°	70.7°	77.20°	−16.7°	0°	16.7°
Facet 5	80.5°	0°	−16.7°	0°	16.7°	80.74°	84.92°	−16.7°	0°	16.7°

**Table 2:** EMD values between surface A and facets 1–16 of the standard body.

Surface No.	EMD Value to Surface A	Surface No.	EMD Value to Surface A
Facet 1	69.9691	Facet 2	63.3331
Facet 3	51.5347	Facet 4	35.7649
Facet 5	75.5017	Facet 6	78.9820
Facet 7	36.9407	Facet 8	22.9104
Facet 9	52.1091	Facet 10	61.2501
Facet 11	68.0692	Facet 12	52.6523
Facet 13	41.0880	Facet 14	67.0089
Facet 15	62.8452	Facet 16	77.0957

**Table 3:** CPFAO and CPLA scoring.

Stop Distance (cm)	Score	Stop Distance (cm)	Score	Stop Distance (cm)	Score
0~15	0	105~120	35	210~225	70
15~30	5	120~135	40	225~240	75
30~45	10	135~150	45	240~255	80
45~60	15	150~165	50	255~270	85
60~75	20	165~180	55	270~285	90
75~90	25	180~195	60	285~300	95
90~105	30	195~210	65	>300	100

**Table 4:** Test results of CPFAO scenario for new and aged pedestrian targets. Distances between stopping point and theoretical collision point and AEB test scores at different speeds and in different trials.

Target Type	Speed (km/h)	Test No.	Distance (m)	Score (points)
New pedestrian target	10	Test 1	0.43	10
		Test 2	/	/
		Test 3	/	/
	20	Test 1	1.40	45
		Test 2	0	0
		Test 3	0	0
	30	Test 1	0	0
		Test 2	0.09	0
		Test 3	0	0
Aged pedestrian target	10	Test 1	1.93	60
		Test 2	0.54	20
		Test 3	0.98	30
	20	Test 1	0	0
		Test 2	/	/
		Test 3	1.04	30
	30	Test 1	0.72	20
		Test 2	0.53	15
		Test 3	0.33	10

**Table 5:** Test results of CPLA scenario for new and aged pedestrian targets. Distances between stopping point and theoretical collision point and AEB test scores at different speeds and in different trials.

Target Type	Speed (km/h)	Test No.	Distance (m)	Score (Points)
New pedestrian target	10	Test 1	0	0
		Test 2	1.3	40
		Test 3	1.15	30
	15	Test 1	0.23	5
		Test 2	0.05	0
		Test 3	0	0
	20	Test 1	0.2	5
		Test 2	1.3	40
		Test 3	0.45	15
Aged pedestrian target	10	Test 1	0	0
		Test 2	2.42	80
		Test 3	/	/
	15	Test 1	0.01	0
		Test 2	0.59	15
		Test 3	0	0
	20	Test 1	0.49	15
		Test 2	0.54	15
		Test 3	0.12	0

## Data Availability

Data is contained within the article.

## Abbreviations

The following abbreviations are used in this manuscript:

AEB	Automatic Emergency Braking System
BRDF	Bidirectional Reflectance Distribution Function
EMD	Earth Mover’s Distance
RCS	Radar Cross-Section
TDMFSB	Three-Dimensional Multi-Faceted Standard Body
CPFAO	Car-to-Pedestrian Farside Adult with Obstruction
CPLA	Car-to-Pedestrian Longitudinal Adult
ADAS	Advanced Driver Assistance System
V2V	Vehicle-To-Vehicle
UAV	Unmanned Aerial Vehicle
CNY	Chinese Yuan
WP	White Plate
MF	Matching Facet
MeF	Measured Facet

## Appendix A

To facilitate understanding, the annotations in Figure A1a are explained as an illustrative example. The labeling conventions in Figure A1b–d and Figure A2, Figure A3, Figure A4, Figure A5 and Figure A6 are identical to those defined in Figure A1a.

Figure A1a shows the following:

- Grating door time: The moment when the front end of the VUT passes through the grating door, at which time the pedestrian target’s traction system is activated, and the pedestrian target begins to move.
- Warning time: The first moment when the AEB system issues an alert to the driver.
- Stop time: The moment when the VUT comes to a complete stop.
- Pedestrian target displacement: The curve represents the change in displacement of the pedestrian target over time.
- Vehicle displacement: The curve represents the change in displacement of the VUT over time.
- Pedestrian target velocity: The curve represents the change in velocity of the pedestrian target over time.
- Vehicle velocity: The curve represents the change in velocity of the VUT over time.
- Left vertical axis: The velocity axis.
- Right vertical axis: The displacement axis.
- Horizontal axis: The time axis, with the moment the VUT begins to move set as the time-zero point.
- Distance between stop point and crash point: The measured distance between the front end of the vehicle and the anticipated crash point after the VUT comes to a complete stop.
- Distance between alert point and crash point: A combination of the distance difference between the alert point and the stop point and the distance between the stop point and the crash point.
